# The Use of Waivers of Guardian Permission in the United States for Pediatric Digital Health Intervention Research Since 2020: Systematic Review

**DOI:** 10.2196/87047

**Published:** 2026-09-25

**Authors:** Colleen Stiles-Shields, Elyse Shenberger, Ann Bernica, Gabriella Bobadilla, Kandis Fletcher, Jasmine George, Sydney Gordon, Erika L Gustafson, Crystal S Hernandez, Taylor L Hilderbrand, Sarah N Kumi, Christine A Lee, Sarah I Lorduy, Meera Phanse, Lauren M Potthoff, Naomi Quezada, Dale L Smith, Holly J Hudson, Emily E Anderson

**Affiliations:** 1 Department of Psychiatry, Institute for Juvenile Research, University of Illinois Chicago, 1747 W. Roosevelt Road, Chicago, IL, 60618, United States, 1 312-413-1128; 2 College of Medicine, AI.Health4All Center, University of Illinois Chicago, Chicago, IL, United States; 3 College of Medicine Peoria, Peoria campus of the University of Illinois System, Peoria, IL, United States; 4 OSF Children's Hospital of Illinois, Peoria, IL, United States; 5 Department of Clinical Psychology, University of Houston - Clear Lake, Houston, TX, United States; 6 Southern University and Agricultural and Mechanical College, Baton Rouge, LA, United States; 7 Neiswanger Institute for Bioethics and Healthcare Leadership, Loyola University Chicago, Maywood, IL, United States; 8 Department of Psychology, Loyola University Chicago, Chicago, IL, United States; 9 Chicago Medical School, Rosalind Franklin University of Medicine and Science, North Chicago, IL, United States; 10 Department of Psychology and Neuroscience, Baylor University, Waco, TX, United States; 11 Pritzker Department of Psychiatry and Behavioral Health, Lurie Children's Hospital, Chicago, IL, United States; 12 Department of Psychiatry and Behavioral Sciences, Feinberg School of Medicine, Northwestern University, Chicago, IL, United States; 13 Library of the Health Sciences - Rockford, University of Illinois Chicago, Chicago, IL, United States

**Keywords:** guardian permission, consent, digital health interventions, youth, teens, ethics, PRISMA, Preferred Reporting Items for Systematic Reviews and Meta-Analyses

## Abstract

**Background:**

Digital health interventions (DHIs) offer scalable ways to reach youth. While DHI research often poses minimal risk, guardian permission is typically required for minors to participate. As a result, requiring guardian permission may unintentionally obstruct safe, high-quality research and delay its translation.

**Objective:**

This review aims to explore practices related to guardian permission. This study systematically reviewed randomized controlled trials of DHIs with minors from the United States published between January 1, 2020, and June 17, 2026.

**Methods:**

PubMed, Embase, PsycINFO, and ACM Digital Library were searched; Cochrane Library and Web of Science yielded only duplicates and were therefore excluded. Included studies had to be: conducted in the United States, randomized controlled trials evaluating DHIs for pediatric mental and/or behavioral health, written in English, and peer-reviewed. Studies were excluded if participation was decided at the school level, included caregivers or pregnant teens as participants, or had a mean sample age of more than 18 years. Two coders independently screened and extracted data, with a third, unique reviewer resolving discrepancies. Risk of bias was assessed using the revised Cochrane risk of bias tool for randomized trials (RoB 2). A narrative framework was used to identify themes. Inverse variance-weighted linear regressions and logistic regressions were conducted to compare studies with and without permission waivers.

**Results:**

Of 7170 screened studies, 49 met inclusion criteria (21 used guardian permission waivers; 28 required permission). The most commonly reported rationale for waiver use was participant privacy. Logistic regressions showed that studies with waivers were more likely to assess gender beyond the binary of male-female (odds ratio [OR] 4.27, 95% CI 1.22-14.93; *P*=.02) and more likely to recruit online (OR 0.04, 95% CI 0.01-0.17; *P*<.001). Weighted study-level regression revealed significantly lower follow-up retention in studies with waivers (mean 77.03%, SD 12.32%) compared to those requiring guardian permission (mean 88.61%, SD 17.17%; *P*=.005). No significant differences emerged across age, race, ethnicity, assessment of suicidality, sensitive topic assessment, intervention focus, or treatment retention.

**Conclusions:**

Though several frameworks exist for evaluating ethics around waiving guardian permission for minors in research, this is the first review to systematically evaluate research in DHI, which often varies in terms of its level of risk. Although small sample sizes and variable reporting practices limited statistical power, the lack of differences in sample characteristics or study features between studies that used waivers and those that did not suggests that institutional review boards may be differentially interpreting regulations and guidance on waiving guardian permission. These findings likely indicate the need for more evidence to guide risk and benefit evaluations—or more education about the existing evidence base, as well as the risks and potential benefits of pediatric DHI research.

## Introduction

### Background

In the United States, there is an unprecedented youth mental health crisis [1,2], exacerbated by provider shortages [3], recent federal budget cuts limiting access to mental health care, and changes to insurance policies [4]. Youth from systemically excluded communities (eg, lower socioeconomic status and less access to behavioral health services) are particularly impacted [5,6]. Digital health interventions (DHIs) have been posited as a promising avenue for addressing these barriers given their accessibility and scalability [7,8]. Indeed, most youth own a digital device [9]. Yet, access to digital devices and the internet does not necessarily equate to access to evidence-based DHIs. Despite growing up alongside smartphones, evidence-based digital health tools rarely reach young people in their daily lives [10].

Guardian permission is generally required for youth in the United States younger than the age of 18 years to participate in research. Given that guardian permission requires disclosure about the nature of the study, necessitating guardian involvement may differentially impact or exclude particular youth, especially if the research involves sensitive or stigmatized topics. There is growing evidence that some adolescents would prefer not to involve their caregivers in decisions about research on topics such as sexuality, sexual health, and depression [11-13]. Many US states allow minors as young as 12 years of age to receive behavioral and/or mental health treatment without guardian consent [14], but it is not always clear if these state laws cover research [15]. Requiring guardian permission for research on adolescent behavioral and mental health limits the participation of certain subgroups and skews research findings. It may also increase adolescents’ vulnerability by limiting access to free, confidential, and potentially beneficial treatment [16].

There is evidence that mandating guardian permission for research may lead to limited participation for certain populations. For example, studies recruiting minors who (1) may identify as lesbian, gay, bisexual, transgender, queer or questioning, intersex, asexual, aromantic (LGBTQIA+); (2) are at risk for or have experienced a sexually transmitted infection; or (3) engage in substance use or other risky behaviors, require their adolescent participants to weigh the pros and cons of asking their guardians for permission. Indeed, one study found that nearly 40% of LGBTQIA+ identified youth reported they would not participate in research if guardian permission was required, for fear of being “outed” to unsupportive family members [13]. As such, forcing adolescents to disclose aspects of their identity to guardians to receive evidence-based intervention may carry more risks than participating in the research itself [14,17]. Further, this requirement may also skew findings. A meta-analysis of research involving youth who engage in substance use or other risky behaviors found significant differences between studies that required guardian permission and those that had a waiver of permission. Indeed, studies without a waiver had decreased substance use disclosure, fewer participants with minoritized racial or ethnic identity, and fewer male participants [18].

### Rationale

Given this evidence, there is debate about, and variable practices regarding, the requirement for guardian permission in digital health research focused on serving adolescents. The intended purpose of guardian permission is to ensure that adolescents are not pressured into participating in research, that they understand the research they participate in—including the risks and benefits of that research—and that their rights and interests are protected during the research. Historical injustices (eg, Willowbrook Hepatitis study [19]; 1939 Monster Study [20]) have led to conservative research oversight practices, particularly for research with minors. There is a general consensus that guardians have their children’s best interests in mind and are thus in the best position to make decisions on their behalf. This, coupled with the widely held belief that minors do not have the cognitive capacity to make certain decisions for themselves [21], is reflected in the legal age of 18 years for consent to many activities. However, there is ample evidence that many youth younger than 18 years have the cognitive resources to provide meaningful informed consent for certain health care services and research participation [22]. Most DHI research presents no greater than minimal risk, and certain risks (eg, risk of self-harm) can be mitigated without guardian permission if other safeguards are in place [23]. Institutional review boards (IRBs) have the authority to grant a waiver of guardian permission for a particular research project [24], but they use this authority inconsistently [25]—despite 2018 revisions to the Common Rule aimed at making “right-size” requirements for minimal risk social, behavioral, and educational research [26].

Some of these difficulties may stem from the fact that regulations for informed consent in research studies were not developed with current DHI research in mind. Indeed, many of the informed consent regulations were grounded in the context of in-person, medical environments. While DHI may deliver evidence-based skills and tools, similar to in-person care, a key difference is how DHI can reach youth in the moment and in their daily lives. More than 90% of teens report accessing the internet regularly, a trend that is consistent across diverse demographic groups (ie, gender, race, ethnicity, and household income) [27]. DHI has been established as an effective, accessible, and scalable option for delivering mental health screening and intervention to those traditionally excluded from care [7,8]. Yet, current regulations do not meet the flexibility and accessibility of DHI as a delivery mechanism. This issue is particularly pertinent for certain groups, such as adolescents who identify as LGBTQIA+, who turn to online spaces for information and support when traditional, in-person communities may be exclusive, hard to reach, and/or discriminatory [12]. Requiring guardian permission for these adolescents—and therefore their identity, behavior, or symptom disclosure to caregivers—might unintentionally prevent DHI research and tools from reaching the very youth for which they were designed. Moreover, misinformation is readily available on the internet and freely accessed by adolescents without guardian controls. In this milieu, requiring guardian permission to participate in research on evidence-based tools is arguably an overly conservative approach. As such, requiring guardian permission may ultimately do more harm than good. In sum, this barrier can restrict participation, create barriers to safe and rigorous research, and hinder the translation of findings into practice. While all adolescents might benefit from access to effective DHIs, restrictions on research can deepen inequities by further limiting the participation of youth from marginalized communities who are more often excluded from research.

### Objectives

While waiving guardian permission for minors may promote inclusion of more young people in trials, research on this practice has been largely theoretical [14,16,17,28,29] and/or narrowly focused on specific minoritized populations (eg, LGBTQIA+ teens and teens engaged in risky behavior [13,18,29,30]). This focus has limited our understanding of the use of waivers of guardian permission in digital contexts. Previous reviews related to this topic include (1) reviews of ethics and practices for obtaining consent or permission for youth in countries outside the United States [22,31]; (2) reviews and synthesis from the perspectives of youth participating in research [32,33]; (3) reviews focused on adolescent decisional capacities for consent [22,34-36]; and (4) a meta-analysis of effects of requiring guardian permission for research on adolescent risk behaviors, where studies included 13 school-based settings and one clinic-based setting [18]. To the best of our knowledge, this is the first review to systematically review reports of randomized controlled trials (RCTs) of DHI with adolescents to better understand the use of waivers of guardian permission for study participation and the potential impact on sample characteristics and participant retention. For the current review, studies were limited to those conducted since January 1, 2020 (in which DHI research markedly increased due to COVID-19 social distancing requirements) and those conducted in the United States, given international differences in requirements for consent and age of majority. The focus of this review was on sample characteristics, methodological choices in assessment, intervention characteristics, and assessment retention for studies that did and did not report using a waiver of guardian permission.

## Methods

### Eligibility Criteria

To be included in the review, studies had to (1) contain US samples (as consent regulations vary internationally) with an average age <18 years; (2) examine the intervention or assessment of a mental or behavioral health condition via digital tools (ie, DHI as active arm of RCT); (3) report primary outcomes of an RCT; (4) be published after January 1, 2020 (in which DHI research markedly increased due to COVID-19 social distancing requirements); (5) be written in English; and (6) be published in a peer-reviewed journal. As the primary outcome examined in this review was whether or not a waiver of guardian permission was used, studies that (1) were randomized at the school level (eg, a digital tool was implemented in a classroom setting without teen assent); (2) included parents as study participants (as there would be no opportunity for waiving guardian permission); or (3) included pregnant teens (as state laws vary regarding their legal rights), were excluded. Technical validation papers, conference abstracts, secondary data analyses, review papers, and studies with participants living outside of the United States and/or primarily the age of 18 or older were also excluded.

### Information Sources

This systematic review was conducted and reported following the PRISMA (Preferred Reporting Items for Systematic Reviews and Meta-Analyses) checklist in Checklist 1 [37] and was registered prior to data extraction in PROSPERO [38]. The search was conducted according to the PRISMA-S (Preferred Reporting Items for Systematic Reviews and Meta-Analyses literature search extension) [39], and the checklist is available in Checklist 2. The original search was conducted in March 2024 and was rerun and updated in June 2026. A literature search was originally run separately in the following databases: PubMed, Embase (Elsevier), and PsycINFO (EBSCOhost). Originally, searches were also run in the Cochrane Library (Wiley) and Web of Science (Thomson Reuters); however, these did not provide any unique titles and, as such, were excluded from the search. In response to the peer review process, an additional database was searched in April 2026 and again in June 2026: ACM Digital Library. As ACM Digital Library does not have mechanisms for searching by publication type and is also not capable of handling complex searches, the librarian coauthor adapted the search strategy. Namely, 2 separate searches with the same search terms but using 2 different field tags (“title” and “abstract”) were run. The results consisted of the searches above; no study registries were searched, nor were online resources and browsing, citation searches, author contacting, or other methods used.

### Search Strategies

Both controlled vocabulary (ie, MeSH terms) and keywords relating to adolescents, mental health, and the digital environment were searched. No restrictions were placed on the search in terms of language (ie, an unrestricted search followed by English-language screening); however, a limit was placed on the date of publication (2020 or later), and efforts were made to exclude studies that took place outside of the United States. As this study is only concerned with RCTs, and RCT field tags and verbiage vary among databases, the use of validated search strategies from ISSG (InterTASC Information Specialists’ Sub-Group) Search Filters was deemed necessary [40-42]. The search strategy was not based on prior work nor peer review process. Rather, the search strategy was developed collaboratively and iteratively by 2 authors (CS-S and EEA) and a trained medical librarian (HKH). The initial search was conducted by a trained librarian (HKH) in February 2024. To update the findings over time, an additional search was conducted by a trained librarian (HKH) in June 2026. A reproducible search strategy for each database is listed in Multimedia Appendix 1.

### Selection Process

An online systematic review software, Covidence (Veritas Health Innovation Ltd), facilitated study selection [43]. At each stage (ie, abstract screening, full-text screening, and data extraction), 2 unique coders evaluated the studies. Fourteen coders screened 7170 titles and abstracts for inclusion, in duplicate; 12 coders screened 217 full-text manuscripts for inclusion, in duplicate; and 11 coders extracted data from 49 studies [44-90], in duplicate. Inclusion discrepancies at all stages were resolved through consensus with a third reviewer. Covidence’s auto-mark tool for ineligibility was used (ie, labeled as “Non-RCT”; 1202 marked as ineligible); no other machine learning algorithms were used to prioritize screening.

### Data Collection Process

Reviewer teams extracted data independently and in duplicate from each eligible study using an extraction form housed in Covidence [43]. This form was piloted and calibrated with all reviewers prior to formal data extraction. Discrepancies about inclusion were resolved by discussion, and a third reviewer was brought in to provide consensus as necessary.

### Data Items: Outcomes

Outcome data are listed here, including how data were requested to be extracted by the coding team, in parentheses. A waiver of guardian permission was determined as being used via (1) an explicit mention of obtaining and/or using a waiver of guardian permission in the text; and/or (2) the authors stating that they received teen or minor assent without accompanying information about guardian permission and/or consent. Studies were deemed to have required guardian permission (ie, not using a waiver of guardian permission) if guardian permission and/or consent was detailed in the paper. Primary data outcomes collected were: (1) whether a waiver of guardian permission was used (coded as yes, no, or unsure, with author’s description included for clarification) and if a waiver was used, rationale for use of a waiver (yes, no, or unsure; recorded verbatim from manuscript and categorized by first author [eg, privacy]); (2) sample characteristics: sample size (enter number), age (enter range and average), sex (enter number of males and females), racial and ethnic identity reporting (enter as reported), lingual status of the sample (coded as English-speaking participants only; bilingual participants [specify languages], non–English-speaking participants [specify languages]); (3) assessments: whether gender was assessed beyond the binary (coded as yes, no, or unsure, with author’s description included for clarification), assessment of suicidality (coded as yes, no, or unsure, with author’s description included for clarification), assessment of other sensitive topics (eg, mental health and sexual activities; coded as yes, no, or unsure, with author’s description included for clarification), number of completed assessments at end of treatment and follow-up (entered based on CONSORT [Consolidated Standards of Reporting Trials] chart); and (4) intervention characteristics and methodologies: population or target of intervention (eg, teens with depression), recruitment source (entered verbatim; eg, online and clinic referral), digital tool use (coded as applied: app, webpage, texting, passive tracking, and other [specify]), duration of intervention (eg, single interaction and repeated use).

### Data Items: Other Variables

Other data items collected were: (1) description of the assent process (detailed verbatim from manuscript); (2) intervention and control arm descriptions (detailed verbatim from manuscript; ensured met inclusion criteria); and (3) reported use of intervention (detailed verbatim from manuscript).

### Study Risk of Bias Assessment

Risk of bias (quality assessment) was also assessed at data extraction, using the revised Cochrane risk of bias tool for randomized trials (RoB 2) [91], which uses 9 standard criteria to evaluate randomized trials.

### Effect Measures

Due to the variability of methods and outcome measurements, a meta-analytic approach was deemed inappropriate to assess what are ultimately differences in study characteristics (see Checklist 3 for the Synthesis Without Meta-analysis [SWiM] reporting items [92]). Rather, both a systematic narrative framework and the use of inverse variance-weighted linear regressions and logistic regression were used to explore categorical study characteristics, and inverse variance-weighted regression of logit-transformed proportions was used to explore retention and other proportions across study types. For the systematic narrative framework, results were classified under “Studies Using a Waiver of Guardian Permission” or “Studies Requiring Guardian Permission.” The systematic narrative synthesis included sample characteristics, sensitive topic assessments, and focus of intervention. These categories were selected prior to data collection, as they would provide key insights into how DHIs have been recently studied—and with which populations, with and without a waiver of guardian permission. To contextualize the findings, rationale for using a waiver of guardian permission was also extracted, if included in the paper.

### Synthesis Methods

#### Overview

All phases of study screening and extraction occurred and were housed in Covidence [43]. All studies were eligible for synthesis following the removal of duplicates by Covidence. To prepare for synthesis, participant characteristics that were presented as percentages (eg, % of females) were tabulated to total numbers. No visual displays of individual studies occurred during synthesis. Due to limited missing data, there was low risk of bias due to missing results (ie, a focus on sample characteristics and methodological decisions led to a low incidence of missing results for the review).

#### Statistical Synthesis Methods

Inverse variance-weighted regression and logistic regressions were used to explore if studies using a waiver of guardian permission were more or less likely than those that required guardian permission to have: (1) recruited online versus in-person; (2) an older average sample age; (3) assessed gender beyond the binary (ie, beyond male-female); (4) enrolled a more diverse sample based on race; (5) included reports of sample ethnicity; (6) enrolled a more diverse sample based on ethnicity; (7) assessed suicidality; (8) assessed other sensitive topics (eg, sexuality and substance use); (9) intervened on more sensitive topics (ie, substance use, sexual health, nonsuicidal self-injury [NSSI], and minoritized identity); (10) used a single interaction or longer interaction period for the primary intervention; (11) higher treatment retention; and (12) higher follow-up retention. Due to the exploratory nature of these analyses, no sensitivity or certainty analyses were conducted. Analyses were conducted in R (version 4.3.2; R Core Team) [93,94].

## Results

### Study Selection

#### Overview

**Figure 1.**
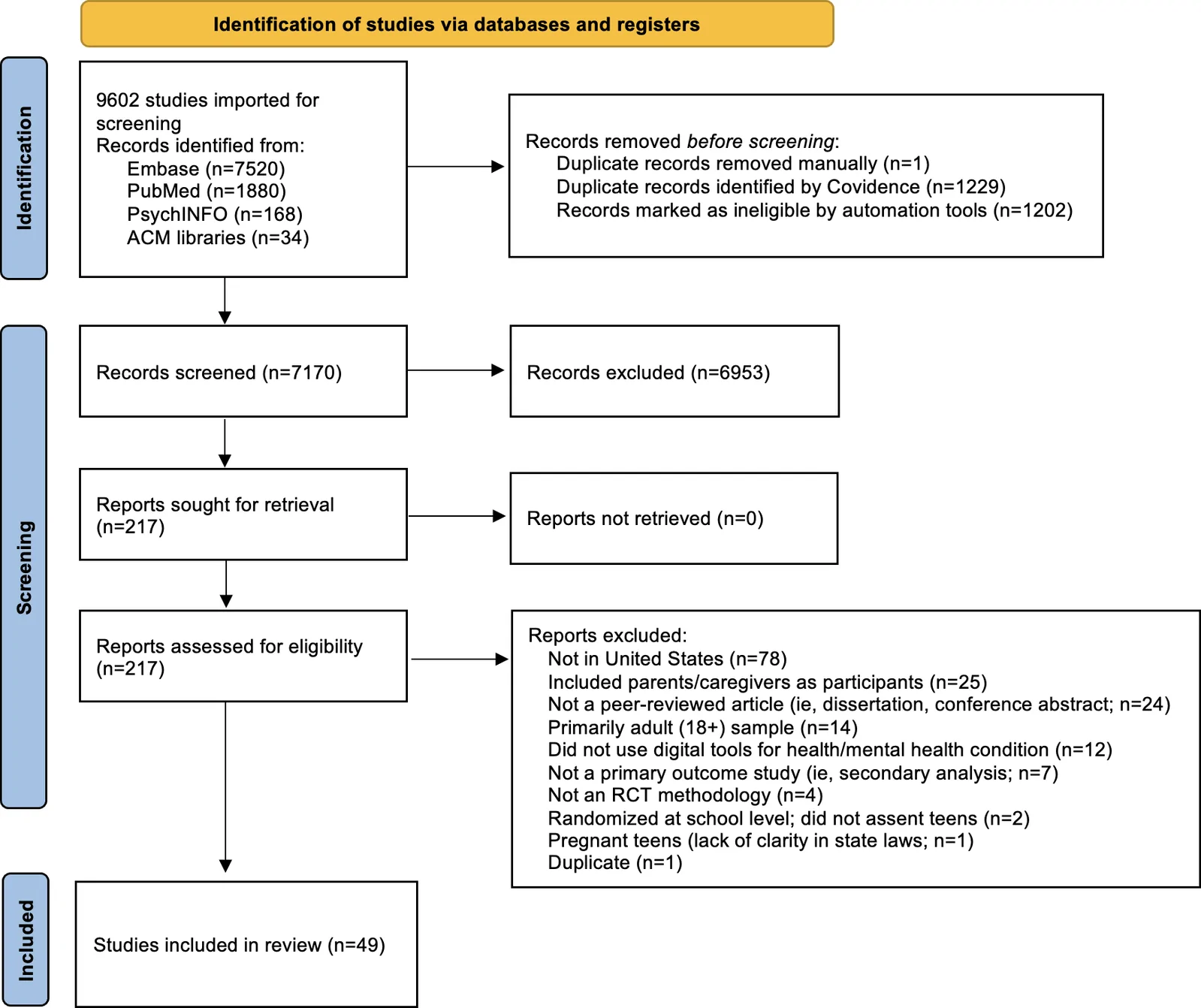
PRISMA (Preferred Reporting Items for Systematic Reviews and Meta-Analyses) flow diagram outlining steps for study inclusion for randomized controlled trials for digital health interventions in a US-based, adolescent population. Adapted from Page et al [37]. RCT: randomized controlled trial.

Following the removal of duplicates identified by Covidence, 7170 titles and abstracts were reviewed by 2 independent reviewers. A total of 217 full-text articles were reviewed by 2 independent reviewers for inclusion, with 49 articles selected for data extraction. Refer to Figure 1 for the PRISMA flow diagram. Of the 49 studies included for extraction [44-90,95,96], 21 used a waiver of guardian permission [44-62,95,96] and 28 required guardian permission [63-90]. All 49 of these studies were used in inferential analyses except where noted.

#### Excluded Studies

Studies excluded were those (1) with a sample outside of the United States [97]; (2) with a sample with an average age >18 years [98]—including those that included caregivers as participants (which inherently implies guardian permission and awareness of participation; eg, [99]); (3) without DHI as an active arm of the RCT [100]; (4) that did not report primary outcomes of an RCT [101]; (5) that were not written in English; (6) that included pregnant teens, as state laws were not specified in terms of emancipation [102]; and (7) were not published in a peer-reviewed journal (eg, conference abstract [103]; protocol paper [104]).

### Study Characteristics

Table 1 presents the study characteristics, 21 of which reported using a waiver of guardian permission [44-62,95,96] and 28 of which reported obtaining guardian permission [63-90].

**Table 1.** Study characteristics.

Study (year)	Title	Condition or experience	Waiver of guardian permission used
Amsalem and Martin (2022) [95]	Reducing depression-related stigma and increasing treatment seeking among adolescents: randomized controlled trial of a brief video intervention	Stigma toward depression	Yes
Bauermeister et al (2022) [45]	An identity-affirming web application to help sexual and gender minority youth cope with minority stress: pilot randomized controlled trial	Minority stress	Yes
Benoit et al (2025) [57]	Climate change hopefulness, anxiety, and behavioral intentions among adolescents: randomized controlled trial of a brief “selfie” video intervention	Climate anxiety	Yes
Cavazos-Rehg et al (2026) [58]	Acceptability, feasibility, and preliminary effectiveness of a mobile app for teens with or at high risk for eating disorders: a three-arm pilot randomized controlled trial comparing self-help, guided self-help, and guided self-help with a social networking component	Eating disorders	Yes
Dobias et al (2021) [46]	An online, single-session intervention for adolescent self-injurious thoughts and behaviors: results from a randomized trial	Nonsuicidal self-injury in adolescents	Yes
Egan et al (2021) [47]	Feasibility of a web-accessible game-based intervention aimed at improving help seeking and coping among sexual and gender minority youth: results from a randomized controlled trial	Help-seeking and coping	Yes
France et al (2020) [44]	A randomized controlled trial of a tablet-based intervention to address predonation fears among high school donors	Fear surrounding blood donation	Yes
Glass et al (2024) [59]	Effectiveness of the myPlan Teen App, a digital healthy relationship and safety planning intervention with adolescents aged 15‐17 years	Domestic violence victimization	Yes
Graham et al (2024) [60]	A vaping cessation text message program for adolescent e-cigarette users: a randomized clinical trial	Nicotine vaping (e-cigarette use)	Yes
Gryczynski et al (2021) [48]	Computer- vs. nurse practitioner-delivered brief intervention for adolescent marijuana, alcohol, and sex risk behaviors in school-based health centers.	Substance use and risky sexual behaviors	Yes
Kutok et al (2021) [49]	A cyberbullying media-based prevention intervention for adolescents on Instagram: pilot randomized controlled trial	Cyberbullying	Yes
Manvelian et al (2026) [61]	Project Relate: a randomized clinical trial of a digital single-session romantic competence intervention	Romantic competence	Yes
Martin et al (2022) [51]	Destigmatizing perceptions about Black adolescent depression: randomized controlled trial of brief social contact-based video interventions	Depression	Yes
Martínez-García et al (2023) [50]	Crush: a randomized trial to evaluate the impact of a mobile health app on adolescent sexual health	Sexual and reproductive health	Yes
Rinehart et al (2020) [52]	Acceptability and efficacy of a sexual health texting intervention designed to support adolescent females	Sexual health	Yes
Rushing et al (2021) [53]	Efficacy of an mHealth intervention (brave) to promote mental wellness for American Indian and Alaska Native teenagers and young adults: randomized controlled trial	Mental health and resilience	Yes
Schleider et al (2022) [54]	A randomized trial of online single-session interventions for adolescent depression during COVID-19	Depression	Yes
Schnall et al (2022) [96]	Efficacy of MyPEEPS mobile, an HIV prevention intervention using mobile technology, on reducing sexual risk among same-sex attracted adolescent males: a randomized clinical trial	Prophylactic use and HIV prevention	Yes
Shen et al (2023) [55]	Randomized evaluation of an online single-session intervention for minority stress in LGBTQ+ adolescents	Minority stress	Yes
Smith et al (2025) [62]	Randomized trial of a digital single-session intervention for body image and mood concerns among LGBTQ+ adolescents	Body image and mood concerns	Yes
Van Meter and Agrawal (2022) [56]	LovesCompany: evaluating the safety and feasibility of a mental health-focused online community for adolescents.	Peer support in the context of mental health	Yes
Bowen-Jallow et al (2021) [63]	Wearable activity tracking device use in an adolescent weight management clinic: a randomized controlled pilot trial	Obesity	No
Bruzzese et al (2021) [64]	The development and preliminary impact of CAMP Air: a web-based asthma intervention to improve asthma among adolescents	Asthma	No
Carson et al (2025) [82]	A multiple technology–based physical activity intervention for Latina adolescents: results from the Chicas Fuertes randomized controlled trial	Moderate-vigorous physical activity	No
Cioffi and Lubetzky (2023) [65]	BOXVR versus guided YouTube boxing for stress, anxiety, and cognitive performance in adolescents: a pilot randomized controlled trial	Stress, anxiety, and cognitive performance	No
Clement et al (2024) [66]	Feasibility, usability, and acceptability of MobileCoach-Teen: a smartphone app-based preventative intervention for risky adolescent drinking behavior.	Risky drinking behavior	No
Crosby et al (2020) [90]	Improving self-management in adolescents with sickle cell disease	Sickle cell disease	No
Doumas et al (2020) [67]	Efficacy of the eCHECKUP TO GO for high school seniors: sex differences in risk factors, protective behavioral strategies, and alcohol use	Alcohol use	No
Doumas et al (2021) [68]	A randomized controlled trial of the eCHECKUP TO GO for high school seniors across the academic year	Alcohol use	No
Dupuis et al (2023) [69]	Food allergy management for adolescents using behavioral incentives: a randomized trial	Adolescents with food allergies	No
Eaton et al (2020) [81]	Text messaging adherence intervention for adolescents and young adults with chronic kidney disease: pilot randomized controlled trial and stakeholder interviews	Medication adherence for young people with chronic kidney disease	No
Garber et al (2025) [83]	A randomized controlled trial of an online mindfulness program for adolescents at risk for internalizing problems	Trait negative affectivity	No
Goldstein et al (2025) [84]	Bridging gaps in care following hospitalization for suicidal adolescents: As Safe as Possible (ASAP) and BRITE App	Suicidality	No
Hoag et al (2022) [70]	Distracting through procedural pain and distress using virtual reality and guided imagery in pediatric, adolescent, and young adult patients: randomized controlled trial	Perception of pain and anxiety	No
Iyer et al (2023) [71]	Impact of the Heartfulness program on loneliness in high schoolers: randomized survey study	Loneliness (mental health)	No
Kaushal et al (2022) [72]	A text messaging intervention with financial incentive for adolescents with type 1 diabetes	Type I diabetes	No
Marsch et al (2021) [73]	Evaluating the effectiveness of a web-based program (POP4Teens) to prevent prescription opioid misuse among adolescents: randomized controlled trial	Opioid misuse	No
McCrimmon et al (2024) [85]	Evaluation of a brief online sexual health program for adolescents: a randomized controlled trial	Sexual health	No
Paraskeva et al (2025) [86]	Evaluating the effectiveness of a Roblox Video Game (Super U Story) in improving body image among children and adolescents in the United States: randomized controlled trial	Body image	No
Peake et al (2024) [74]	Preliminary efficacy of a digital intervention for adolescent depression: randomized controlled trial	Depression	No
Richardson et al (2021) [75]	Electronic health risk behavior screening with integrated feedback among adolescents in primary care: randomized controlled trial	Health risk behaviors	No
Sadasivam et al (2026) [87]	Feasibility and engagement of a peer-driven mobile intervention for adolescent e-cigarette cessation: cluster randomized pilot study	Nicotine vaping (e-cigarette use)	No
Sayegh et al (2022) [76]	Randomized pilot trial of praise text messages to improve medication adherence among adolescents and young adults with liver transplants	Medical adherence post liver transplants	No
Sayegh et al (2024) [88]	Randomized pilot trial of cell phone support to improve medication adherence among adolescents and young adults with chronic health conditions	Medication adherence	No
Schleider et al (2020) [77]	Randomized trial of a single-session growth mind-set intervention for rural adolescents’ internalizing and externalizing problems	Internalizing and externalizing problems	No
Schwinn et al (2021) [78]	Longitudinal outcomes of a smartphone application to prevent drug use among Hispanic youth	Adolescent substance use	No
Steinberg^a^ et al (2024) [89]	Is there a place for cognitive restructuring in brief, self-guided interventions? Randomized controlled trial of a single-session, digital program for adolescents	Cognitive restructuring skills	No
Thomas-Smith et al (2022) [79]	Electronic screening for adolescent risk behaviors in the emergency department: a randomized controlled trial	Risk behaviors in adolescents	No
Webb et al (2022) [80]	Which adolescents are well-suited to app-based mindfulness training? A randomized clinical trial and data-driven approach for personalized recommendations	Rumination	No

a In this study, caregivers could opt their child out of participating, rather than providing direct permission.

### Risk of Bias in Studies

The RoB 2 [91] was used to evaluate the included studies. Figure S1 in Multimedia Appendix 2 presents the traffic light outcomes, created using the *robvis* R package [105]. “Low Risk” was the most frequent rating across the criteria used by the RoB 2: (1) risk of bias arising from the randomization process (38/49, 77.55%) [44-53,55-60,62-64,66-69,74-82,84-86,88,89,96]; (2) risk of bias due to deviations from the intended interventions (39/49, 79.59%) [45-49,51-57,59,60,62,64,66-68,71-79,81-84,86-90,95,96]; (3) missing outcome data (49/49, 100%) [44-90,95,96]; (4) risk of bias in measurement outcomes (49/49, 100%) [44-90,95,96]; (5) risk of bias in selection of the reported result (49/49, 100%) [44-90,95,96]; and (6) overall risk of bias (31/49, 63.27%) [45-49,51-53,55-57,59,60,62,64,66-68,74-79,81,82,84,86,88,89,96]. Reasons for a rating other than “Low Risk” were related primarily to a lack of clarity in the paper, which often reflected the reality of recruiting a national sample online—often with anonymity. This lack of clarity resulted in ratings of “Some concerns” (occurring a total of 11/245 times, 4.49%) or “No information” (occurring a total of 10/245 times, 4.08%); any rating of either of these led to an overall risk of bias rating of “Some concerns” (18/49, 36.73%).

### Results of Individual Studies and Syntheses

#### Studies Using a Waiver of Guardian Permission

##### Sample Characteristics

Samples that used a waiver of guardian permission (n=21) [44-62,95,96] ranged from 84 to 2452 youth participants (mean 746.00, SD 640.35) and included youth with an average age of 16.19 (SD 0.69) years. Two studies did not report age beyond the inclusion criteria [53,54]. Three-quarters (16/21, 76.19%) assessed and reported gender beyond the binary of sex [45-47,49,51,53-62]. While all studies reported racial identity, one study did not report ethnicity for the sample [49]. Only one study (1/21, 4.76%) reported Middle Eastern and North African (MENA) as an ethnic identity [45]. Across studies, the percentage of participants who identified with a minoritized racial identity was 48.01% (7532/15,688), and 19.60% (3261/16,634) of participants identified as Hispanic or Latiné or MENA. No studies indicated linguistic diversity (ie, no description of materials in multiple languages, nor assessment of linguistic status outside of English). Participants were primarily recruited online [45-47,49-51,53-62,95,96], with some studies also recruiting participants through in-person interactions at schools (ie, high school blood drives) [44], health sites (ie, school-based health center [48], federally qualified community health centers [52], Indian Health Service [53]), or community-based organizations [96].

##### Sensitive Topic Assessments

Sensitive topic assessment included items that might be associated with social stigmas, including both behaviors (eg, substance use) and identities (eg, sexual orientation). About a quarter of studies assessed suicidal ideation (6/21, 28.57%) [46,47,54,56,59,61]. Beyond assessment of suicidal ideation, most studies assessed at least one other sensitive topic (17/21, 80.95%) [45-50,52-56,59-62,95,96], including: sexual orientation (14/21, 66.67%) [45-47,49,50,52-56,60-62,96], socioeconomic status or insurance status (3/21, 14.29%) [49,52,62], violence exposure (3/21, 14.29%) [49,59,60], comorbidities (3/21, 14.29%) [60-62], substance use (3/21, 14.29%) [53,60,95], languages spoken at home (1/21, 4.76%) [55], and academic performance (1/21, 4.76%) [55].

##### Focus of Intervention

The focus of the interventions was primarily mental health (13/21, 61.90%) [45-48,50,51,54-58,61,62] and minoritized identity support (7/21, 33.33%) [45,47,48,52-54,62]. Other foci of intervention included sexual health (4/21, 19.05%) [44,52,61,96], substance use (3/21, 14.29%) [53,60,95], and medical condition management (2/21, 9.52%) [47,49]. To deliver the intervention, the active arms of the RCTs used the following: (1) webpages [46,47,54-56,61,62]; (2) apps [44,48-50,58,59,96]; (3) texting or SMS [52,53,60]; (4) videos [51,95], including TikTok (TikTok Pte Ltd) reels [57]; (5) web-based apps [45]; (6) coaching [58]; and (7) social media [58]. Roughly half of the interventions were designed to be used in a single interaction [44,46,48,51,54,55,57,61,62,95]; whereas the remainder were designed for interactions across weeks to months [45,47,49,50,52,53,56,58-60,96].

##### Rationale for Using a Waiver

Thirteen (13/21, 61.90%) studies provided at least one rationale for obtaining a waiver of guardian permission [45-50,52,54,55,57,58,60,62]. Table 2 presents the rationales. The most frequently reported rationales were (1) to maintain participant privacy, including situations in which caregiver knowledge of youth participation could affect participants’ safety (eg, youth who have not shared their gender or sexual identity with a caregiver) or they are unlikely to disclose a behavior to a guardian (eg, e-cigarette use) [45,47,50,54,55,60,62]; and (2) state legislation that allows for adolescents to pursue mental or behavioral health care without guardian involvement [46,48,50,52,55]. Other rationales included: (1) receiving IRB approval for waiving guardian permission [46,54,57,60], (2) minimal risk to participant safety [46,55,61], (3) wishing to minimize stigma and increase participant candor around sensitive topics [46,48], (4) recommendations from national, professional bodies (eg, Society of Adolescent Medicine) [49,50], and (5) being in accordance with the Common Rule [49,106]. One study did not provide a rationale for using a waiver of guardian permission, instead describing in detail how age verification was completed for teen participants [56].

**Table 2.** Rationales for waiving guardian permission.

Waiver rationale (n=15)	Frequency, n (%)	Study
Participant privacy	7 (46.67)	Bauermeister et al (2022) [45] Egan et al (2021) [47] Graham et al (2024) [60] Martínez-Garcia et al (2023) [50] Schleider et al (2022) [54] Shen et al (2023) [55] Smith et al (2025) [62]
State legislation for adolescents to pursue mental or behavioral health care without guardian involvement	5 (33.33)	Dobias et al (2021) [46] Gryczynski et al (2021) [48] Martínez-Garcia et al (2023) [50] Rinehart et al (2020) [52] Shen et al (2023) [55]
Receiving institutional review board approval for waiving guardian permission	4 (26.67)	Benoit et al (2025) [57] Dobias et al (2021) [46] Graham et al (2024) [60] Schleider et al (2022) [54]
Minimal risk	3 (20)	Cavazos-Rehg et al (2026) [58] Dobias et al (2021) [46] Shen et al (2022) [55]
Minimize stigma and increase participant candor around sensitive topics	2 (13.33)	Dobias et al (2021) [46] Gryczynski et al (2021) [48]
Recommendations from national, professional societies	2 (13.33)	Kutok et al (2021) [49] Martínez-Garcia et al (2023) [50]
In accordance with the Common Rule	1 (6.67)	Kutok et al (2021) [49]

### Studies Requiring Guardian Permission

#### Sample Characteristics

Samples requiring guardian permission (n=28) [63-90], one of which offered guardians the option to opt their child out of participation [89], ranged in size from 29 to 1617 youth participants (mean 245.04, SD 319.43), with the average age of participants being 15.35 (SD 1.66) years. Two (2/28, 7.14%) studies did not report age beyond the inclusion criteria [71,75]. Fewer than half of the samples (12/28) assessed and reported gender beyond the binary of sex. One (1/28, 3.57%) [65] study did not report participants’ racial identities, and one study only reported the number of participants who identified as “Non-Hispanic White” (1/28, 3.57%) [87]. Five (5/28, 17.86%) studies did not report participants’ ethnicity, with none reporting MENA as an identity [65,70,71,81,90]. Across studies, 41.61% (2751/6612) of participants identified with a minoritized racial identity, and 23.02% (1515/6582) of participants identified as Hispanic or Latiné. One study reported recruiting bilingual participants (ie, English and Spanish) [82] and one study reported examining outcomes for a subsection of the sample that learned English as a second language [85]; no other linguistic diversity was reported. Participants were primarily recruited through in-person interactions or invitations at health sites (ie, weight management clinic [63], Children’s Hospital of Philadelphia at Penn Medicine [69], nephrology clinic [81], hematology/oncology clinics [70,90], diabetes clinic [72], primary care [75], transplant clinic [76], emergency department [79], inpatient unit [84], and direct invitations from health care provider [88]), schools [64-68,77,78,85,87,89], or community-based organizations [64,78,80]. Eight (8/28, 28.57%) studies reported using online recruitment methods, often in conjunction with in-person efforts [66,71,73,74,78,82,83,86].

#### Sensitive Topic Assessments

Five (5/28, 7.14%) studies assessed suicidal ideation [74,75,79,80,84]. Beyond assessment of suicidal ideation, most studies assessed other sensitive topics (26/28, 92.86%) [63-75,77-89], including: substance use (7/28, 25%) [68,70,73,74,81,84,87], comorbidities (6/28, 21.43%) [63,69,83,84,86,89], socioeconomic status or insurance status (4/28, 14.29%) [84,86,88,89], academic performance (3/28, 10.71%) [69,73,76], languages spoken at home (1/28, 3.57%) [82], and sexual orientation (1/28, 3.57%) [85].

#### Focus of Intervention

The focus of the interventions was primarily mental health (10/28, 35.71%) [64,67,71,75,78,80,83,84,86,89], medical condition management (9/28, 32.14%) [65,66,72,75,77,79,82,88,90], and substance use (6/28, 21.43%) [68,70,73,74,81,87]. Other foci of intervention included minoritized identity support (2/28, 7.14%) [63,71], sexual health (2/28, 7.14%) [74,85], and NSSI (2/28, 7.14%) [69,84]. In terms of intervention delivery, the active arms of the RCTs used the following: (1) webpages [64,67-69,71,73,75,77,82,83,85,89]; (2) texting or SMS [64,67-69,71,72,75,76,82,87,88]; (3) apps [63,66,74,75,78,80,84,90]; (4) virtual reality [65,70]; (5) passive tracking tools [63,82]; (6) coaching or peer support [82-84,87,88]; (7) social media [82]; (8) video game [86]; and (9) an electronic health record tool [79]. Twenty of the interventions (20/28, 71.43%) were designed for interactions across weeks to months [63-66,69-74,76,78,80-84,87,88,90], whereas the other nearly a quarter of the studies (8/28, 28.57%) were deployed through a single interaction [67,68,75,77,79,85,86,89].

### Comparisons: Waiver of Guardian Permission Versus Requiring Guardian Permission

Many of the analyses were underpowered due to the low number of studies included in the review, as well as differences in reporting practices. Therefore, we report these findings as exploratory in nature. Logistic regressions predicting whether gender was assessed (ie, beyond the binary of male-female) were significant (n=49, odds ratio [OR] 4.27, 95% CI 1.22-14.93; *P*=.02), which indicated that studies that used a waiver of guardian consent had 4 times greater odds of assessing and reporting gender. Recruitment methodologies were significantly associated with waiver status, such that studies that primarily recruited through in-person settings (eg, a specific clinic) had 96% lower odds of having used a waiver of guardian permission relative to those that recruited online (n=49, OR 0.04, 95% CI 0.01-0.17; *P*<.001). We found no evidence to indicate differences based on waiver status in the assessment of suicidal ideation (n=49, OR 1.84, 95% CI 0.48-7.12; *P*=.38) or sensitive topics (n=49, OR 0.71, 95% CI 0.16-3.23; *P*=.66), or primary focus of intervention (ie, mental health [*P*=.07]; substance use [*P*=.53]; sexual health [*P*=.23]; NSSI [*P*=.99]; minoritized identity support [*P*=.06]; medical condition [*P*=.08]).

Significant differences were detected in the inverse variance-weighted regression of logit-transformed proportions assessing differences in participant retention at follow-up, across the 37 studies [45,47-50,52-56,58-63,65,67,68,70,71,73,75-81,84,86-90,95,96] providing this information, between studies using a waiver (mean 77.03%, SD 12.32%) and those requiring guardian permission (mean 88.61%, SD 14.17%; n=37, *P*=.005). However, there was no significant difference in treatment retention prior to follow-up between studies using a waiver (mean 91.46%, SD 0.09%) and studies requiring guardian permission (mean 93.92%, SD 0.08%; n=42, *P*=.96) across these same studies. We also found no significant differences based on duration of the intervention (ie, single interaction vs longer duration; n=49, *P*=.18). There was also no evidence to suggest differences in the average age of participants (mean [waiver] 16.19%, SD 0.69%; mean [consent] 15.35%, SD 1.66%; n=45, *P*=.08), the percentage of participants identifying with a minoritized racial identity (mean [waiver] 27.82%, SD 21.33%; mean [consent] 20.80%, SD 11.79%; n=44, *P*=.48) or minoritized ethnicity (mean [waiver] 23.91%, SD 16.23%; mean [consent] 27.72%, SD 30.23; n=38, *P*=.96).

## Discussion

### Principal Findings

Within the context of the increased shift to digital interventions following the COVID-19 pandemic, this is the first systematic review of its kind to characterize DHI RCTs that used a waiver of guardian permission with participants who were minors (ie, younger than the age of 18 years) in the United States. The most commonly reported rationale for obtaining a waiver of guardian permission was to maintain the privacy of minor participants. This rationale included situations in which participant safety may be impacted by caregiver knowledge of participation, such as a teenager who has not shared their sexual orientation identity. Methodologically, studies that used a waiver were more likely to assess gender beyond the binary and to recruit participants online (as opposed to clinic-based recruitment). Further, studies that used a waiver had lower follow-up retention compared with studies that obtained guardian permission. There was no evidence to suggest significant differences in other methodological choices or sample makeup based on the use of a waiver of guardian permission.

### Interpretation

Despite a small sample size, 3 significant differences emerged between studies that did and did not use a waiver of guardian permission. First, gender, rather than biological sex (ie, male vs female), was more likely to be inclusively assessed and/or reported by studies that used a waiver. Given that sexual orientation was frequently noted as a rationale for a need for adolescent privacy through a waiver, such studies and research teams may be more attuned to the appropriate assessment and representation of gender [107]. Second, studies that used a waiver were more likely to recruit online—likely due to the fact that these studies were recruiting national samples. Third, studies that required guardian permission were much more likely to use in-person—often, in clinic recruitment; these studies also reported higher follow-up retention rates. A likely explanation is that such studies included frequent and ongoing in-person contact, which supported better follow-up retention. Indeed, such a difference in retention may be more related to the recruitment site and the fact that participants had some in-person contact with study staff, at least at the time of consent and enrollment, rather than being influenced by the requirement for guardian permission.

There is robust discussion in the research literature and proposed frameworks for determining when it is ethically acceptable to waive guardian permission for adolescent minors’ research participation [32,33,108,109]. However, these discussions have not adequately considered research on DHI—research that takes place in an online virtual environment, and which vary in terms of their level of risk [110]. While there may be some consensus, for example, on situations in which requiring guardian permission may not be feasible, would reduce participation by adolescents in ways that impact scientific integrity (eg, representativeness of the sample), or risk harm to participants [108,111], the results of the current review imply variability in use of waivers of guardian permission in DHI research. Indeed, given a lack of differences in sample characteristics or study features between studies that used waivers and those that did not, one hypothesis as to why this might be is that IRBs may be differentially interpreting regulations and guidance on waiving guardian permission. While differences in IRB decisions and practices may be reasonable, given the flexibility in federal guidance [112-114], if this hypothesis is true, it could also be an indication of a need for more evidence to guide risk and benefit evaluation or more education about existing evidence and the risks and potential benefits of DHI research. It is also reasonable to assume that some of these studies that required guardian permission could have been granted a waiver on legal and ethical solid ground (ie, it may be justified that “parental or guardian permission is not a reasonable requirement to protect the subjects” (46.408c) [115]).

A waiver or alteration to informed consent (ie, 46.116f3 [116]) for adult participants requires that “the research could not practicably be carried out without the requested waiver or alteration” [116]. This is generally interpreted to mean that there is an unusually high burden on researchers to obtain consent, for example, because of distance or reachability. Risk of harm to participants if their identity becomes known outside the research is often used as a justification for waiving consent in research with adults [117], although the regulations do not specifically speak to this. While there is no practicability requirement for waiving guardian permission spelled out in Subpart D [118], some IRBs invoke this to generally require guardian permission when parents are identifiable and “reachable” for informed consent. In our sample, more studies that required guardian permission used in-person recruitment methods—and specifically, recruited from clinics. These adolescents very likely had a guardian with them when they were approached for participation and thus obtaining guardian consent was “practicable” (feasible).

The Guidelines on the Inclusion and Protection of Adolescent Minors and Young Adults in Health Research (2025) from the Society for Adolescent Health and Medicine (SAHM) provide a useful framework for making determinations about waiving guardian permission [108]. First, given evidence that the capacity to provide informed consent for research is present by age 14, in order to respect evolving capacity, guardian permission should not be required for any research study that poses no more than minimal risk for adolescents aged 14 years and older. Second, guardian permission should not be required if it is impractical and otherwise ethically unnecessary. Third, guardian permission should not be required for research participants if it compromises adolescents’ right to confidential health care or preventative services. Fourth, guardian permission to research should not be required if it threatens adolescents’ safety and well-being. Finally, there may be certain studies for which requiring guardian permission might be “otherwise ethically problematic.” While the SAHM guidance does not speak specifically to DHI research [108], using these 5 criteria, many more studies included in our sample would be eligible for a waiver of guardian permission.

### Limitations of Evidence

Multiple limitations of the current evidence should be considered. First, it is unclear which, if any, studies requested a waiver of guardian permission and were denied by their IRB. Second, it is unknown on what grounds requests for waivers may have been denied, and we have incomplete information on reasons some waivers were granted. Third, it is unclear which, if any, studies might have been granted a waiver by their IRB but did not request one. These details would provide more insight into IRB decision-making and how they interpret the flexibility to grant waivers of guardian permission consistent with federal regulations. Finally, statistical power for inferential comparisons was limited due to the available sample size. This also limits the ability to adjust for multiple comparisons to control type I errors in this research without further increases in type II errors due to additional loss of power. As such, inferential results should be interpreted with caution.

### Limitations of Review Processes

The findings from this review should be considered in light of specific limitations. First, our search strategy (Multimedia Appendix 1) excluded specific countries in an attempt to identify US-based studies (eg, NOT (“Canada”[Mesh] OR “Europe”[Mesh]...”). As such, it is possible that multinational studies that included US cohorts were omitted, thereby limiting potentially relevant data. Additionally, our search strategy did not include citation searches, which will be particularly important moving forward for reviews that are not date-limited, such as the current review. Second, this review focused on the initial “burst” of DHI studies following social distancing recommendations made during the COVID-19 pandemic (ie, 2020‐2026). It is very likely that there is and will be a growing body of pediatric DHI RCTs that do and do not use a waiver of guardian permission. Future research should continue to review and monitor the impacts of both choices. Third, data collected for this review were based on what was reported in peer-reviewed publications. While this decision is in line with a reliance on research transparency in academic publishing—“a cornerstone of credible research” [119], there is a possibility of misclassification bias in this review. Finally, the included studies were conducted with US samples. While adolescent autonomy is a universal construct, age of majority and guardian permission regulations vary across countries. While ethical justifications for using waivers of guardian permission in research may be universal across borders, the actual practice will be heavily influenced by national legal requirements, and thus our results may not be generalizable to DHI studies with adolescents in other countries.

### Implications

Though several frameworks exist for evaluating the ethical acceptability of waiving guardian permission for research, no reviews have focused on the actual use of waivers of guardian permission in pediatric DHI. As such, this review contributes to the literature by characterizing the use of waivers of guardian permission in pediatric DHI research. Our review suggests that waivers of guardian permission for DHI research are granted inconsistently across studies and institutions and that the use of waivers may have an impact on participant retention—laying the groundwork for future research and education in this area.

### Conclusions

Approaches to recruitment, informed consent and assent, and guardian permission in DHI research should respect adolescents’ developing autonomy, support appropriate research participation, minimize risk, and ensure representativeness of samples. Empirical research is needed to develop an evidence base to support investigator and IRB decision-making about waivers of parental permission. Standard reporting of whether or not waivers were requested, whether or not the IRB granted the request for the waiver, and if denied, on what grounds, would be useful in improving understanding of how IRBs interpret federal guidance and make decisions about waivers. Documenting and reporting actual harms and other critical incidents (eg, reports of intended self-harm that required intervention) that occur in DHI research with adolescents could also inform best practices for requesting waivers, allowing waivers, and implementing additional protections that protect adolescents in DHI research while also promoting inclusion.

## Supplementary material

10.2196/87047 Multimedia Appendix 1 Search strategy.

10.2196/87047 Multimedia Appendix 2 The Revised Cochrane Risk-of-Bias tool for randomized trials.

10.2196/87047 Checklist 1 PRISMA 2020 main and abstract checklists.

10.2196/87047 Checklist 2 PRISMA-S checklist.

10.2196/87047 Checklist 3 SWiM checklist.

## Abbreviations

CONSORT: Consolidated Standards of Reporting Trials
DHI: digital health intervention
IRB: institutional review board
ISSG: InterTASC Information Specialists' Sub-Group
LGBTQIA+: lesbian, gay, bisexual, transgender, queer or questioning, intersex, asexual/aromantic
MENA: Middle Eastern and North African
NSSI: nonsuicidal self-injury
OR: odds ratio
PRISMA: Preferred Reporting Items for Systematic Reviews and Meta-Analyses
PRISMA-S: Preferred Reporting Items for Systematic Reviews and Meta-Analyses literature search extension
RCT: randomized controlled trial
RoB2: revised Cochrane risk of bias tool for randomized trials
SAHM: Society for Adolescent Health and Medicine
SWiM: Synthesis Without Meta-analysis

## References

[1] Merikangas KR, He J ping, Burstein M (2011). Service utilization for lifetime mental disorders in U.S. adolescents: results of the National Comorbidity Survey-Adolescent Supplement (NCS-A). J Am Acad Child Adolesc Psychiatry.

[2] Murthy VH (2021). Protecting youth mental health: the US Surgeon General’s Advisory. https://www.hhs.gov/sites/default/files/surgeon-general-youth-mental-health-advisory.pdf.

[3] Whitney DG, Peterson MD (2019). US national and state-level prevalence of mental health disorders and disparities of mental health care use in children. JAMA Pediatr.

[4] Update on cuts to Medicaid funding and restrictions to Medicaid access. American Psychological Association.

[5] Lu W, Todhunter-Reid A, Mitsdarffer ML, Muñoz-Laboy M, Yoon AS, Xu L (2021). Barriers and facilitators for mental health service use among racial/ethnic minority adolescents: a systematic review of literature. Front Public Health.

[6] Murthy VH (2022). The mental health of minority and marginalized young people: an opportunity for action. Public Health Rep.

[7] Lattie EG, Stiles-Shields C, Graham AK (2022). An overview of and recommendations for more accessible digital mental health services. Nat Rev Psychol.

[8] Linardon J, Torous J, Firth J, Cuijpers P, Messer M, Fuller-Tyszkiewicz M (2024). Current evidence on the efficacy of mental health smartphone apps for symptoms of depression and anxiety. A meta-analysis of 176 randomized controlled trials. World Psychiatry.

[9] Anderson A (2023). Teens, social media and technology 2023. Pew Research Center.

[10] Psihogios AM, Lane-Fall MB, Graham AK (2022). Adolescents are still waiting on a digital health revolution: accelerating research-to-practice translation through design for implementation. JAMA Pediatr.

[11] Schleider JL, Smock A, Ahuvia IL (2025). State parental consent law and treatment use among adolescents with depression. JAMA Pediatr.

[12] Moskowitz DA, Macapagal K, Mongrella M, Pérez-Cardona L, Newcomb ME, Mustanski B (2020). What if my dad finds out!?: assessing adolescent men who have sex with men’s perceptions about parents as barriers to PrEP uptake. AIDS Behav.

[13] Cwinn E, Cadieux C, Crooks CV (2021). Who are we missing? The impact of requiring parental or guardian consent on research with lesbian, gay, bisexual, trans, two-spirit, queer/questioning youth. J Adolesc Health.

[14] Kiperman S, DeLong G, Varjas K, Meyers J, Lytle MC, Sprott RA (2021). Supporting Gender Identity and Sexual Orientation Diversity in K-12 Schools.

[15] Wendler D (2024). Pediatric research without parental permission. J Pediatr.

[16] Bauman LJ, Mellins CA, Klitzman R (2020). Whether to waive parental permission in HIV prevention research among adolescents: ethical and legal considerations. J Law Med Ethics.

[17] Flores D, McKinney R, Arscott J, Barroso J (2018). Obtaining waivers of parental consent: a strategy endorsed by gay, bisexual, and queer adolescent males for health prevention research. Nurs Outlook.

[18] Liu C, Cox RB, Washburn IJ, Croff JM, Crethar HC (2017). The effects of requiring parental consent for research on adolescents’ risk behaviors: a meta-analysis. J Adolesc Health.

[19] Rothman DJ (2017). The Willowbrook Wars.

[20] Silverman FH (1988). The “monster” study. J Fluency Disord.

[21] Roth-Cline M, Nelson RM (2013). Parental permission and child assent in research on children. Yale J Biol Med.

[22] Mathews B (2023). Adolescent capacity to consent to participate in research: a review and analysis informed by law, human rights, ethics, and developmental science. Laws.

[23] Stevens K, Thambinathan V, Hollenberg E (2021). Core components and strategies for suicide and risk management protocols in mental health research: a scoping review. BMC Psychiatry.

[24] Research with children FAQS. US Department of Health and Human Services.

[25] Shah S, Whittle A, Wilfond B, Gensler G, Wendler D (2004). How do institutional review boards apply the federal risk and benefit standards for pediatric research?. JAMA.

[26] (2009). Federal Policy for the Protection of Human Subjects ('Common Rule'). US Department of Health and Human Services.

[27] Atske S (2025). Teens and social media fact sheet. http://www.pewresearch.org/internet/fact-sheet/teens-and-social-media-fact-sheet/.

[28] Stablein T, Jacobs SH, Bass LE, Kinney DA (2011). The Well-Being, Peer Cultures and Rights of Children.

[29] Smith AU, Schwartz SJ (2019). Waivers of parental consent for sexual minority youth. Account Res.

[30] Nelson KM, Carey MP, Fisher CB (2019). Is guardian permission a barrier to online sexual health research among adolescent males interested in sex with males?. J Sex Res.

[31] MacNeill L, MacNeill AL, Doucet S, Luke A, Goudreau A (2024). Obtaining consent for research on risky behaviours among adolescents in Canada: a scoping review. J Empir Res Hum Res Ethics.

[32] Crane S, Broome ME (2017). Understanding ethical issues of research participation from the perspective of participating children and adolescents: a systematic review. Worldviews Evid Based Nurs.

[33] Ren Y, Tang S, Ji G (2026). Experiences and perspectives of children and adolescents participating in pediatric clinical trials: a qualitative evidence synthesis. Arch Public Health.

[34] Parmigiani G, Benevento M, Solarino B (2025). Decisional capacity to consent to treatment in children and adolescents: a systematic review. Psychiatry Res.

[35] Brown HL, Underhill K, Gradus JL, Nelson KM (2026). History of minor consent laws for mental health treatment in the US. JAMA Health Forum.

[36] Spriggs M (2023). Children and bioethics: clarifying consent and assent in medical and research settings. Br Med Bull.

[37] Page MJ, McKenzie JE, Bossuyt PM (2021). The PRISMA 2020 statement: an updated guideline for reporting systematic reviews. Rev Esp Cardiol (Engl Ed).

[38] The role of waiving guardian consent in pediatric digital health intervention research since 2020: systematic review. PROSPERO.

[39] Rethlefsen ML, Kirtley S, Waffenschmidt S (2021). PRISMA-S: an extension to the PRISMA statement for reporting literature searches in systematic reviews. Syst Rev.

[40] Lefebvre, Glanville J, Briscoe S Cochrane Handbook for Systematic Reviews of Interventions Version 651.

[41] (2020). Randomized controlled trials/controlled clinical trials: a cut and paste search strategy adapted from CADTH for Ovid PsycINFO. University of Alberta.

[42] (2024). HSLS LibGuides.

[43] Covidence.

[44] France CR, France JL, Kowalsky JM (2020). A randomized controlled trial of a tablet-based intervention to address predonation fears among high school donors. Transfusion.

[45] Bauermeister J, Choi SK, Bruehlman-Senecal E (2022). An identity-affirming web application to help sexual and gender minority youth cope with minority stress: pilot randomized controlled trial. J Med Internet Res.

[46] Dobias ML, Schleider JL, Jans L, Fox KR (2021). An online, single-session intervention for adolescent self-injurious thoughts and behaviors: results from a randomized trial. Behav Res Ther.

[47] Egan JE, Corey SL, Henderson ER (2021). Feasibility of a web-accessible game-based intervention aimed at improving help seeking and coping among sexual and gender minority youth: results from a randomized controlled trial. J Adolesc Health.

[48] Gryczynski J, Mitchell SG, Schwartz RP (2021). Computer- vs. nurse practitioner-delivered brief intervention for adolescent marijuana, alcohol, and sex risk behaviors in school-based health centers. Drug Alcohol Depend.

[49] Kutok ER, Dunsiger S, Patena JV (2021). A cyberbullying media-based prevention intervention for adolescents on Instagram: pilot randomized controlled trial. JMIR Ment Health.

[50] Martínez-García G, Ewing AC, Olugbade Y, DiClemente RJ, Kourtis AP (2023). Crush: a randomized trial to evaluate the impact of a mobile health app on adolescent sexual health. J Adolesc Health.

[51] Martin A, Calhoun A, Páez J, Amsalem D (2022). Destigmatizing perceptions about Black adolescent depression: randomized controlled trial of brief social contact–based video interventions. Child Psychology Psychiatry.

[52] Rinehart DJ, Leslie S, Durfee MJ (2020). Acceptability and efficacy of a sexual health texting intervention designed to support adolescent females. Acad Pediatr.

[53] Rushing SC, Kelley A, Bull S (2021). Efficacy of an mHealth Intervention (BRAVE) to promote mental wellness for American Indian and Alaska native teenagers and young adults: randomized controlled trial. JMIR Ment Health.

[54] Schleider JL, Mullarkey MC, Fox KR (2022). A randomized trial of online single-session interventions for adolescent depression during COVID-19. Nat Hum Behav.

[55] Shen J, Rubin A, Cohen K (2023). Randomized evaluation of an online single-session intervention for minority stress in LGBTQ+ adolescents. Internet Interv.

[56] Van Meter A, Agrawal N (2022). LovesCompany: evaluating the safety and feasibility of a mental health-focused online community for adolescents. J Child Adolesc Ment Health.

[57] Benoit L, R Lowe S, Thomas I, Amsalem D, Martin A (2025). Climate change hopefulness, anxiety, and behavioral intentions among adolescents: randomized controlled trial of a brief “selfie” video intervention. Child Adolesc Psychiatry Ment Health.

[58] Cavazos‐Rehg PA, Fitzsimmons‐Craft EE, Kasson E (2026). Acceptability, feasibility, and preliminary effectiveness of a mobile app for teens with or at high risk for eating disorders: a three‐arm pilot randomized controlled trial comparing self‐help, guided self‐help, and guided self‐help with a social networking component. Intl J Eating Disorders.

[59] Glass N, Bloom T, Alexander KA (2024). Effectiveness of the myPlan Teen app, a digital healthy relationship and safety planning intervention with adolescent aged 15-17 years. J Adolesc Health.

[60] Graham AL, Cha S, Jacobs MA (2024). A vaping cessation text message program for adolescent e-cigarette users: a randomized clinical trial. JAMA.

[61] Manvelian A, Sotomayor I, Ahuvia I, Bibby ES, Davila J, Schleider J (2026). Project Relate: a randomized clinical trial of a digital single-session romantic competence intervention. Int J Behav Dev.

[62] Smith AC, Ahuvia IL, Zapata JP, Cohen KA, Graham AK, Schleider JL (2025). Randomized trial of a digital single-session intervention for body image and mood concerns among LGBTQ+ adolescents. Behav Res Ther.

[63] Bowen-Jallow K, Nunez-Lopez O, Wright A (2021). Wearable activity tracking device use in an adolescent weight management clinic: a randomized controlled pilot trial. J Obes.

[64] Bruzzese JM, George M, Liu J (2021). The development and preliminary impact of CAMP Air: a web-based asthma intervention to improve asthma among adolescents. Patient Educ Couns.

[65] Cioffi R, Lubetzky AV (2023). *BOXVR* versus guided YouTube boxing for stress, anxiety, and cognitive performance in adolescents: a pilot randomized controlled trial. Games Health J.

[66] Clement A, Ravet M, Stanger C, Gabrielli J (2024). Feasibility, usability, and acceptability of MobileCoach-Teen: a smartphone app-based preventative intervention for risky adolescent drinking behavior. J Subst Use Addict Treat.

[67] Doumas DM, Esp S, Turrisi R, Bond L, Flay B (2020). Efficacy of the eCHECKUP TO GO for high school seniors: sex differences in risk factors, protective behavioral strategies, and alcohol use. J Stud Alcohol Drugs.

[68] Doumas DM, Esp S, Turrisi R, Bond L (2021). A randomized controlled trial of the eCHECKUP to GO for high school seniors across the academic year. Subst Use Misuse.

[69] Dupuis R, Feuerstein-Simon R, Brown-Whitehorn TF (2023). Food allergy management for adolescents using behavioral incentives: a randomized trial. Pediatrics.

[70] Hoag JA, Karst J, Bingen K, Palou-Torres A, Yan K (2022). Distracting through procedural pain and distress using virtual reality and guided imagery in pediatric, adolescent, and young adult patients: randomized controlled trial. J Med Internet Res.

[71] Iyer RB, Vadlapudi S, Iyer L (2023). Impact of the Heartfulness program on loneliness in high schoolers: randomized survey study. Appl Psychol Health Well Being.

[72] Kaushal T, Katz LEL, Joseph J (2022). A text messaging intervention with financial incentive for adolescents with type 1 diabetes. J Diabetes Sci Technol.

[73] Marsch LA, Moore SK, Grabinski M, Bessen SY, Borodovsky J, Scherer E (2021). Evaluating the effectiveness of a web-based program (POP4Teens) to prevent prescription opioid misuse among adolescents: randomized controlled trial. JMIR Public Health Surveill.

[74] Peake E, Miller I, Flannery J, Chen L, Lake J, Padmanabhan A (2024). Preliminary efficacy of a digital intervention for adolescent depression: randomized controlled trial. J Med Internet Res.

[75] Richardson L, Parker EO, Zhou C, Kientz J, Ozer E, McCarty C (2021). Electronic health risk behavior screening with integrated feedback among adolescents in primary care: randomized controlled trial. J Med Internet Res.

[76] Sayegh C, Im D, Moss IK, Urquiza R, Patel S, Thomas DW (2022). Randomized pilot trial of praise text messages to improve medication adherence among adolescents and young adults with liver transplants. Pediatr Transplant.

[77] Schleider JL, Burnette JL, Widman L, Hoyt C, Prinstein MJ (2020). Randomized trial of a single-session growth mind-set intervention for rural adolescents’ internalizing and externalizing problems. J Clin Child Adolesc Psychol.

[78] Schwinn TM, Fang L, Hopkins J, Pacheco AR (2021). Longitudinal outcomes of a smartphone application to prevent drug use among Hispanic youth. J Stud Alcohol Drugs.

[79] Thomas-Smith S, Klein EJ, Strelitz B (2022). Electronic screening for adolescent risk behaviors in the emergency department: a randomized controlled trial. West J Emerg Med.

[80] Webb CA, Swords CM, Lawrence HR, Hilt LM (2022). Which adolescents are well-suited to app-based mindfulness training? A randomized clinical trial and data-driven approach for personalized recommendations. J Consult Clin Psychol.

[81] Eaton C, Comer M, Pruette C, Psoter K, Riekert K (2020). Text messaging adherence intervention for adolescents and young adults with chronic kidney disease: pilot randomized controlled trial and stakeholder interviews. J Med Internet Res.

[82] Carson JR, Greenstadt E, Olivera B (2025). A multiple technology-based physical activity intervention for Latina adolescents: results from the Chicas Fuertes randomized controlled trial. JMIR Mhealth Uhealth.

[83] Garber J, Chavira DA, Adam EK (2025). A randomized controlled trial of an online mindfulness program for adolescents at risk for internalizing problems. J Consult Clin Psychol.

[84] Goldstein TR, Kennard BD, Porta G (2025). Bridging gaps in care following hospitalization for suicidal adolescents: As Safe As Possible (ASAP) and BRITE app. J Am Acad Child Adolesc Psychiatry.

[85] McCrimmon J, Widman L, Javidi H, Brasileiro J, Hurst J (2024). Evaluation of a brief online sexual health program for adolescents: a randomized controlled trial. Health Promot Pract.

[86] Paraskeva N, Haywood S, Anquandah J (2025). Evaluating the effectiveness of a Roblox video game (Super U Story) in improving body image among children and adolescents in the United States: randomized controlled trial. J Med Internet Res.

[87] Sadasivam RS, Stevens EM, Wijesundara J (2026). Feasibility and engagement of a peer-driven mobile intervention for adolescent e-cigarette cessation: cluster randomized pilot study. JMIR Form Res.

[88] Sayegh CS, MacDonell KK, Iverson E (2024). Randomized pilot trial of cell phone support to improve medication adherence among adolescents and young adults with chronic health conditions. BMC Digit Health.

[89] Steinberg JS, Fitzpatrick OM, Khurana S (2024). Is there a place for cognitive restructuring in brief, self-guided interventions? Randomized controlled trial of a single-session, digital program for adolescents. J Clin Child Adolesc Psychol.

[90] Crosby LE, Hood A, Kidwell K (2020). Improving self-management in adolescents with sickle cell disease. Pediatr Blood Cancer.

[91] Sterne JAC, Savović J, Page MJ (2019). RoB 2: a revised tool for assessing risk of bias in randomised trials. BMJ.

[92] Campbell M, McKenzie JE, Sowden A (2020). Synthesis Without Meta-analysis (SWiM) in systematic reviews: reporting guideline. BMJ.

[93] R Core Team (2023). The R project for statistical computing. R Project.

[94] The Comprehensive R Archive Network. The Comprehensive R Archive Network (CRAN).

[95] Amsalem D, Martin A (2022). Reducing depression-related stigma and increasing treatment seeking among adolescents: randomized controlled trial of a brief video intervention. J Child Psychol Psychiatry.

[96] Schnall R, Kuhns LM, Pearson C (2022). Efficacy of MyPEEPS mobile, an HIV prevention intervention using mobile technology, on reducing sexual risk among same-sex attracted adolescent males: a randomized clinical trial. JAMA Netw Open.

[97] Bell I, Arnold C, Gilbertson T (2023). A personalized, transdiagnostic smartphone intervention (Mello) targeting repetitive negative thinking in young people with depression and anxiety: pilot randomized controlled trial. J Med Internet Res.

[98] Chavez LJ, Kelleher K, Slesnick N (2020). Virtual reality meditation among youth experiencing homelessness: pilot randomized controlled trial of feasibility. JMIR Ment Health.

[99] Cushing CC, Bejarano CM, Ortega A, Sayre N, Fedele DA, Smyth JM (2021). Adaptive mHealth intervention for adolescent physical activity promotion. J Pediatr Psychol.

[100] Yarger J, Gutmann-Gonzalez A, Borgen N, Romero J, Decker MJ (2024). In the Know: a cluster randomized trial of an in-person sexual health education program integrating digital technologies for adolescents. J Adolesc Health.

[101] Hollenbach JP, Simoneau T, Sun Y (2021). Design, methods, and baseline characteristics of a pilot, randomized, controlled trial of the effects of an electronic monitoring device on medication adherence in children with asthma. Contemp Clin Trials Commun.

[102] Wambach K, Davis AM, Nelson EL (2025). A health behavior and lifestyle intervention pilot trial for childbearing adolescents. Am J Lifestyle Med.

[103] (2020). SAEM Annual Meeting Abstracts. Acad Emerg Med.

[104] Moore KL, Rodwin AH, Gwadz M, Chang DF, Collins LM, Munson MR (2025). A brief engagement intervention adapted for racial and ethnic minority young adults in mental health services: protocol for a pilot optimization trial. JMIR Res Protoc.

[105] McGuinness LA, Higgins JPT (2021). Risk-of-bias VISualization (robvis): an R package and Shiny web app for visualizing risk-of-bias assessments. Res Synth Methods.

[106] (2009). Protection of human subjects. https://www.govinfo.gov/content/pkg/CFR-2016-title45-vol1/pdf/CFR-2016-title45-vol1-part46.pdf.

[107] Keener E (2015). The complexity of gender: it is all that and more….in sum, it is complicated. Sex Roles.

[108] The Society for Adolescent Health and Medicine (2025). Guidelines on the inclusion and protection of adolescent minors and young adults in health research: a position statement of the Society for Adolescent Health and Medicine. J Adolesc Health.

[109] Ruiz-Canela M, Lopez-del Burgo C, Carlos S (2013). Observational research with adolescents: a framework for the management of the parental permission. BMC Med Ethics.

[110] Harrison S, Islam SU, Waseem HM, Epiphaniou G (2025). Digital health interventions use cases: classification and taxonomy development, a scoping review. Digit Health.

[111] (2018). Guidance on ethical considerations in planning and reviewing research studies on sexual and reproductive health in adolescents. World Health Organization.

[112] Abbott L, Grady C (2011). A systematic review of the empirical literature evaluating IRBs: what we know and what we still need to learn. J Empir Res Hum Res Ethics.

[113] Friesen P, Yusof ANM, Sheehan M (2019). Should the decisions of institutional review boards be consistent?. Ethics Hum Res.

[114] Fost N (2011). IRB inconsistency: be careful what you wish for. AJOB Prim Res.

[115] § 46.408 Requirements for permission by parents or guardians and for assent by children. Electronic Code of Federal Regulations (eCFR).

[116] § 46.116 General requirements for informed consent. Electronic Code of Federal Regulations (eCFR).

[117] Tuohy B, Jatres J (2023). Researching those in the shadows: undocumented immigrants, vulnerability, and the significance of research. Am J Bioeth.

[118] Subpart D — additional protections for children involved as subjects in research. US Department of Health and Human Services.

[119] Wedderkopp N, Rutz E (2024). Scientific integrity and transparency in academic writing: the foundation of credible science. Children (Basel).

