# Supplementary material for: The Use of Waivers of Guardian Permission in the United States for Pediatric Digital Health Intervention Research Since 2020: Systematic Review
Source: J Med Internet Res. 2026 Sep 25;28:e87047. doi: 10.2196/87047 (PMC13614224; doi:10.2196/87047)
Supplement: Supplementary file 1 [file jmir-v28-e87047-s001.docx]

| PubMed | (((((("Adolescent"[Mesh] OR "Adolescent*"[tiab] OR "Adolescence"[tiab] OR "teenager*"[tiab] OR "teen*"[tiab] OR "youth*"[tiab] OR "high school"[tiab] OR "Middle school"[tiab] OR "Minors"[Mesh] OR "Minors"[tiab] OR "Minor"[tiab])) AND (("Mental Health"[Mesh] OR "mental health"[tiab] OR "mental disorders"[Mesh] OR "psychiatric disorder"[tiab] OR "psychiatric illness"[tiab] OR "psychiatric disease"[tiab] OR "mental illness"[tiab] OR "behavioral disorder"[tiab] OR "psychiatric diagnosis"[tiab] OR "severe mental illness"[tiab] OR "severe mental disorder"[tiab] OR "mental disorder"[tiab] OR "behavioral health"[tiab] OR "psychiatric rehabilitation"[Mesh] OR "psychosocial rehabilitation"[tiab] OR "mental health recovery"[tiab] OR "psychiatric rehabilitation"[tiab] OR "mental health rehabilitation"[tiab] OR "psychosocial care"[tiab] OR "psychiatric health"[tiab] OR "health behavior"[Mesh] OR "health behavior"[tiab] OR "coping behavior"[tiab] OR "coping skills"[tiab] OR "coping strategies"[tiab] OR "adaptive behaviors"[tiab] OR "coping"[tiab] OR "stress management"[tiab] OR "treatment adherence and compliance"[mesh] OR "treatment adherence"[tiab] OR "therapeutic adherence and compliance"[tiab] OR "therapeutic adherence"[tiab] OR "adherence"[tiab] OR "condition management"[tiab] OR "mental disease"[tiab] OR "behavior disorder"[tiab] OR "patient compliance"[tiab] OR "psychological adjustment"[tiab]))) AND (("online social networking"[Mesh] OR "Online systems"[Mesh] OR "Online"[tiab] OR "internet"[Mesh] OR "World wide web"[tiab] OR "Internet"[tiab] OR "Website"[tiab] OR "web based"[tiab] OR "cell phone"[Mesh] OR "cellular phone"[tiab] OR "mobile telephone"[tiab] OR "mobile phone"[tiab] OR "digital technology"[Mesh] OR "digital technology"[tiab] OR "smartphone"[mesh] OR "smartphone"[tiab] OR "smart phone"[tiab] OR "android*"[tiab] OR "blackberr*"[tiab] OR "iphone*"[tiab] OR "web blog"[tiab] "blog*"[tiab] OR "vlog*"[tiab] OR "chat room"[tiab] OR "chat rooms"[tiab] OR "chatroom*"[tiab] OR "computers"[mesh] OR "computer*"[tiab] OR "cyber space"[tiab] OR "cyberspace"[tiab] OR "digital"[tiab] OR "digital computer"[tiab] OR "internet panel"[tiab] OR "Zoom"[tiab] OR "videoconferenc*"[tiab] OR "telemedicine"[mesh] OR "virtual health"[tiab] OR "virtual medicine"[tiab] OR "ehealth"[tiab] OR "e health"[tiab] OR "mhealth"[tiab] OR "m health"[tiab] OR "online studies"[tiab] OR "internet studies"[tiab] OR "social media"[mesh] OR "social media"[tiab] OR "online survey"[tiab] OR "text messaging"[mesh] OR "short message service"[mesh] OR "text message"[tiab] OR "text*"[tiab] OR "text messaging"[tiab] OR "digital application"[tiab] OR "notification*"[tiab] OR "digital health"[tiab] OR "digital health technology"[tiab] OR "online health"[tiab] OR "digital mental health"[tiab] OR "electronic mail"[mesh] OR "email*"[tiab] OR "e-mail*"[tiab] OR "instant message"[tiab] OR "instant messaging"[tiab] OR "instant messages"[tiab] OR "instant messenger"[tiab] OR "ipad*" OR "mobile applications"[mesh] OR "mobile application"[tiab] OR "mobile app"[tiab] OR "mobile health"[tiab] OR "Smartphone app"[tiab] OR "Smartphone application"[tiab] OR "mobile healthcare"[tiab] OR "computers, handheld"[mesh] OR "tablet computer"[tiab] OR "palmtop computer"[tiab] OR "palm pilot"[tiab] OR "pocket PC"[tiab] OR "PDA"[tiab] OR "PDAS"[tiab] OR "personal digital assistant"[tiab] OR "mobile device"[tiab] OR "podcast*"[tiab] OR "SMS"[tiab] OR "software"[tiab] OR "tablet*"[tiab] OR "telecare"[tiab] OR "telehealth"[tiab] OR "telemedicine"[tiab] OR "telemonitor*"[tiab] OR "telenursing"[tiab] OR "Web"[tiab] OR "wireless technology"[mesh] OR "wireless"[tiab] OR "blogging"[tiab] OR "vlogging"[tiab] OR "wireless communication"[tiab] OR "mobile health application"[tiab]))) AND (("Survey*"[tiab] OR "Survey methods"[tiab] OR "survey methodology"[tiab] OR "baseline survey"[tiab] OR "respondent*"[tiab] OR "randomized response technique"[tiab] OR "nonrespondent*"[tiab] OR "intervention" OR "crisis intervention"[mesh] OR "crisis intervention"[tiab] OR "brief treatment"[tiab] OR "brief intervention"[tiab] OR "Critical incident stress debriefing"[tiab] OR "psychosocial intervention"[mesh] OR "psychosocial intervention"[tiab] OR "psychological intervention"[tiab] OR "psychophysiological assessment"[tiab] OR "interview, psychological"[mesh] OR "psychological interview"[tiab] OR "psychologic interview"[tiab] OR "questionnaire*"[tiab] OR "interview*"[tiab] OR "Surveys and Questionnaires"[Mesh] OR "Internet-Based Intervention"[Mesh] OR "assessment*" OR "online intervention"[tiab] OR "internet intervention"[tiab] OR "internet based intervention"[tiab] OR "web based intervention"[tiab] OR "Digital Intervention"[tiab] OR "Digital Health Intervention"[tiab] OR "ecological momentary assessment"[mesh] OR "ecological momentary assessment"[tiab] OR "IRB"[tiab] OR "Institutional Review Board"[tiab] OR "institutional review"[tiab] OR "intervention study"[tiab] OR "health survey"[tiab] OR "psychologic assessment"[tiab])) AND "United States") OR ((((("Adolescent"[Mesh] OR "Adolescent*"[tiab] OR "Adolescence"[tiab] OR "teenager*"[tiab] OR "teen*"[tiab] OR "youth*"[tiab] OR "high school"[tiab] OR "Middle school"[tiab] OR "Minors"[Mesh] OR "Minors"[tiab] OR "Minor"[tiab])) AND (("Mental Health"[Mesh] OR "mental health"[tiab] OR "mental disorders"[Mesh] OR "psychiatric disorder"[tiab] OR "psychiatric illness"[tiab] OR "psychiatric disease"[tiab] OR "mental illness"[tiab] OR "behavioral disorder"[tiab] OR "psychiatric diagnosis"[tiab] OR "severe mental illness"[tiab] OR "severe mental disorder"[tiab] OR "mental disorder"[tiab] OR "behavioral health"[tiab] OR "psychiatric rehabilitation"[Mesh] OR "psychosocial rehabilitation"[tiab] OR "mental health recovery"[tiab] OR "psychiatric rehabilitation"[tiab] OR "mental health rehabilitation"[tiab] OR "psychosocial care"[tiab] OR "psychiatric health"[tiab] OR "health behavior"[Mesh] OR "health behavior"[tiab] OR "coping behavior"[tiab] OR "coping skills"[tiab] OR "coping strategies"[tiab] OR "adaptive behaviors"[tiab] OR "coping"[tiab] OR "stress management"[tiab] OR "treatment adherence and compliance"[mesh] OR "treatment adherence"[tiab] OR "therapeutic adherence and compliance"[tiab] OR "therapeutic adherence"[tiab] OR "adherence"[tiab] OR "condition management"[tiab] OR "mental disease"[tiab] OR "behavior disorder"[tiab] OR "patient compliance"[tiab] OR "psychological adjustment"[tiab]))) AND (("online social networking"[Mesh] OR "Online systems"[Mesh] OR "Online"[tiab] OR "internet"[Mesh] OR "World wide web"[tiab] OR "Internet"[tiab] OR "Website"[tiab] OR "web based"[tiab] OR "cell phone"[Mesh] OR "cellular phone"[tiab] OR "mobile telephone"[tiab] OR "mobile phone"[tiab] OR "digital technology"[Mesh] OR "digital technology"[tiab] OR "smartphone"[mesh] OR "smartphone"[tiab] OR "smart phone"[tiab] OR "android*"[tiab] OR "blackberr*"[tiab] OR "iphone*"[tiab] OR "web blog"[tiab] "blog*"[tiab] OR "vlog*"[tiab] OR "chat room"[tiab] OR "chat rooms"[tiab] OR "chatroom*"[tiab] OR "computers"[mesh] OR "computer*"[tiab] OR "cyber space"[tiab] OR "cyberspace"[tiab] OR "digital"[tiab] OR "digital computer"[tiab] OR "internet panel"[tiab] OR "Zoom"[tiab] OR "videoconferenc*"[tiab] OR "telemedicine"[mesh] OR "virtual health"[tiab] OR "virtual medicine"[tiab] OR "ehealth"[tiab] OR "e health"[tiab] OR "mhealth"[tiab] OR "m health"[tiab] OR "online studies"[tiab] OR "internet studies"[tiab] OR "social media"[mesh] OR "social media"[tiab] OR "online survey"[tiab] OR "text messaging"[mesh] OR "short message service"[mesh] OR "text message"[tiab] OR "text*"[tiab] OR "text messaging"[tiab] OR "digital application"[tiab] OR "notification*"[tiab] OR "digital health"[tiab] OR "digital health technology"[tiab] OR "online health"[tiab] OR "digital mental health"[tiab] OR "electronic mail"[mesh] OR "email*"[tiab] OR "e-mail*"[tiab] OR "instant message"[tiab] OR "instant messaging"[tiab] OR "instant messages"[tiab] OR "instant messenger"[tiab] OR "ipad*" OR "mobile applications"[mesh] OR "mobile application"[tiab] OR "mobile app"[tiab] OR "mobile health"[tiab] OR "Smartphone app"[tiab] OR "Smartphone application"[tiab] OR "mobile healthcare"[tiab] OR "computers, handheld"[mesh] OR "tablet computer"[tiab] OR "palmtop computer"[tiab] OR "palm pilot"[tiab] OR "pocket PC"[tiab] OR "PDA"[tiab] OR "PDAS"[tiab] OR "personal digital assistant"[tiab] OR "mobile device"[tiab] OR "podcast*"[tiab] OR "SMS"[tiab] OR "software"[tiab] OR "tablet*"[tiab] OR "telecare"[tiab] OR "telehealth"[tiab] OR "telemedicine"[tiab] OR "telemonitor*"[tiab] OR "telenursing"[tiab] OR "Web"[tiab] OR "wireless technology"[mesh] OR "wireless"[tiab] OR "blogging"[tiab] OR "vlogging"[tiab] OR "wireless communication"[tiab] OR "mobile health application"[tiab]))) AND (("Survey*"[tiab] OR "Survey methods"[tiab] OR "survey methodology"[tiab] OR "baseline survey"[tiab] OR "respondent*"[tiab] OR "randomized response technique"[tiab] OR "nonrespondent*"[tiab] OR "intervention" OR "crisis intervention"[mesh] OR "crisis intervention"[tiab] OR "brief treatment"[tiab] OR "brief intervention"[tiab] OR "Critical incident stress debriefing"[tiab] OR "psychosocial intervention"[mesh] OR "psychosocial intervention"[tiab] OR "psychological intervention"[tiab] OR "psychophysiological assessment"[tiab] OR "interview, psychological"[mesh] OR "psychological interview"[tiab] OR "psychologic interview"[tiab] OR "questionnaire*"[tiab] OR "interview*"[tiab] OR "Surveys and Questionnaires"[Mesh] OR "Internet-Based Intervention"[Mesh] OR "assessment*" OR "online intervention"[tiab] OR "internet intervention"[tiab] OR "internet based intervention"[tiab] OR "web based intervention"[tiab] OR "Digital Intervention"[tiab] OR "Digital Health Intervention"[tiab] OR "ecological momentary assessment"[mesh] OR "ecological momentary assessment"[tiab] OR "IRB"[tiab] OR "Institutional Review Board"[tiab] OR "institutional review"[tiab] OR "intervention study"[tiab] OR "health survey"[tiab] OR "psychologic assessment"[tiab])) NOT ("Canada"[Mesh] OR "Europe"[Mesh] OR "Africa"[Mesh] OR "Asia"[Mesh] OR "Australia"[Mesh] OR "South America"[Mesh] OR "Islands"[Mesh] OR "Greenland"[Mesh] OR "Latin America"[Mesh] OR "Central America"[Mesh] OR "Caribbean Region"[Mesh] OR "Mexico"[Mesh]))) AND ((("Randomized Controlled Trial" [Publication Type] OR "controlled clinical trial"[pt] OR "randomized"[tiab] OR "placebo"[tiab]) OR ("clinical trials as topic" [mesh:noexp]) OR (randomly [tiab] OR trial [ti])) NOT (animals [mh] NOT humans [mh])) |
| --- | --- |
| Date limit | 01/01/2020 – 6/17/2026 |
| Embase | ((((('adolescent*':ti,ab OR 'adolescence':ti,ab OR 'high school student*':ti,ab OR 'middle school student*':ti,ab OR 'minor*':ti,ab OR 'teen*':ti,ab OR 'youth*':ti,ab OR 'adolescent'/exp OR 'adolescent' OR 'adolescence'/exp OR 'adolescence' OR 'high school student'/exp OR 'high school student' OR 'middle school student'/exp OR 'middle school student' OR 'minor (person)'/exp OR 'minor (person)') AND ('mental health'/exp OR 'mental disease'/exp OR 'behavior disorder'/exp OR 'severe mental illness'/exp OR 'severe mental disorder'/exp OR 'behavioral health'/exp OR 'psychosocial rehabilitation'/exp OR 'mental health recovery'/exp OR 'psychosocial care'/exp OR 'health behavior'/exp OR 'coping behavior'/exp OR 'coping skills training'/exp OR 'adaptive behavior'/exp OR 'psychological adjustment'/exp OR 'stress management'/exp OR 'patient compliance'/exp OR 'mental health':ti,ab OR 'mental disease':ti,ab OR 'behavior disorder':ti,ab OR 'severe mental illness':ti,ab OR 'severe mental disorder':ti,ab OR 'behavioral health':ti,ab OR 'psychosocial rehabilitation':ti,ab OR 'mental health recovery':ti,ab OR 'psychosocial care':ti,ab OR 'health behavior':ti,ab OR 'coping behavior*':ti,ab OR 'coping skills training':ti,ab OR 'psychological adjustment*':ti,ab OR 'stress management':ti,ab OR 'patient compliance':ti,ab OR 'mental disorder*':ti,ab OR 'psychiatric disorder*':ti,ab OR 'psychiatric illness':ti,ab OR 'psychiatric disease*':ti,ab OR 'mental illness':ti,ab OR 'behavioral disorder*':ti,ab OR 'psychiatric diagnosis':ti,ab OR 'psychiatric rehabilitation':ti,ab OR 'mental health rehabilitation':ti,ab OR 'psychiatric health':ti,ab OR 'coping skills':ti,ab OR 'coping strategies':ti,ab OR 'adaptive behavior*':ti,ab OR 'coping':ti,ab OR 'treatment adherence':ti,ab OR 'therapeutic adherence and compliance':ti,ab OR 'therapeutic adherence':ti,ab OR 'adherence':ti,ab OR 'condition management':ti,ab) AND ('online social network'/exp OR 'online system'/exp OR 'internet'/exp OR 'mobile phone'/exp OR 'digital technology'/exp OR 'smartphone'/exp OR 'iphone'/exp OR 'blogging'/exp OR 'vlogging'/exp OR 'computer'/exp OR 'personal data assistant'/exp OR 'digital computer'/exp OR 'videoconferencing'/exp OR 'telemedicine'/exp OR 'telecare'/exp OR 'telehealth'/exp OR 'mhealth'/exp OR 'social media'/exp OR 'online survey'/exp OR 'text message'/exp OR 'text messaging'/exp OR 'short message service'/exp OR 'digital health'/exp OR 'digital health technology'/exp OR 'e-mail'/exp OR 'instant messaging'/exp OR 'tablet computer'/exp OR 'mobile application'/exp OR 'mobile health application'/exp OR 'mobile health'/exp OR 'smartphone application'/exp OR 'mobile device'/exp OR 'podcast'/exp OR 'software'/exp OR 'telemonitoring'/exp OR 'telenursing'/exp OR 'wireless communicaiton' OR 'online social network*':ti,ab OR 'online system*':ti,ab OR 'internet':ti,ab OR 'mobile phone':ti,ab OR 'digital technology':ti,ab OR 'smartphone':ti,ab OR 'iphone':ti,ab OR 'blogging':ti,ab OR 'vlogging':ti,ab OR 'computer*':ti,ab OR 'personal digital assistant':ti,ab OR 'digital computer*':ti,ab OR 'videoconferencing':ti,ab OR 'telemedicine':ti,ab OR 'telecare':ti,ab OR 'telehealth':ti,ab OR 'mhealth':ti,ab OR 'social media':ti,ab OR 'online survey':ti,ab OR 'text messag*':ti,ab OR 'short message service':ti,ab OR 'digital health':ti,ab OR 'digital health technology':ti,ab OR 'e-mail':ti,ab OR 'instant messag*':ti,ab OR 'tablet computer*':ti,ab OR 'mobile health application':ti,ab OR 'mobile health*':ti,ab OR 'mobile device':ti,ab OR 'podcast':ti,ab OR 'software':ti,ab OR 'telemonitor*':ti,ab OR 'telenursing':ti,ab OR 'wireless communication':ti,ab OR 'online':ti,ab OR 'world wide web':ti,ab OR 'website':ti,ab OR 'web based':ti,ab OR 'cell phone*':ti,ab OR 'cellular phone*':ti,ab OR 'mobile telephone':ti,ab OR 'mobile phone*':ti,ab OR 'smart phone*':ti,ab OR 'android*':ti,ab OR 'blackberr*':ti,ab OR 'web blog':ti,ab OR 'blog':ti,ab OR 'vlog':ti,ab OR 'chat room*':ti,ab OR 'chatroom*':ti,ab OR 'cyber space':ti,ab OR 'cyberspace':ti,ab OR 'digital':ti,ab OR 'internet panel':ti,ab OR 'zoom':ti,ab OR 'virtual health':ti,ab OR 'virtual medicine':ti,ab OR 'ehealth':ti,ab OR 'e health':ti,ab OR 'm health':ti,ab OR 'online stud*':ti,ab OR 'internet stud*':ti,ab OR 'text*':ti,ab OR 'digital application':ti,ab OR 'notification*':ti,ab OR 'online health':ti,ab OR 'digital mental health':ti,ab OR 'electronic mail':ti,ab OR 'email*':ti,ab OR 'ipad':ti,ab OR 'mobile app*':ti,ab OR 'smartphone app*':ti,ab OR 'palmtop computer':ti,ab OR 'palm pilot':ti,ab OR 'pocket pc':ti,ab OR 'pda*':ti,ab OR 'sms':ti,ab OR 'tablet':ti,ab OR 'web':ti,ab OR 'wireless':ti,ab OR 'wireless technology':ti,ab) AND ('questionnaire'/exp OR 'survey'/exp OR 'health survey'/exp OR 'surveys'/exp OR 'survey methodology'/exp OR 'baseline surveys'/exp OR 'randonmized response technique' OR 'intervention study'/exp OR 'crisis intervention'/exp OR 'brief intervention'/exp OR 'psychological intervention'/exp OR 'psychosocial intervention'/exp OR 'psychological interview'/exp OR 'psychophysiologic assessment'/exp OR 'interview'/exp OR 'web-based intervention'/exp OR 'psychologic assessment'/exp OR 'digital intervention'/exp OR 'digital health intervention'/exp OR 'ecological momentary assessment'/exp OR 'institutional review'/exp OR 'questionnaire*':ti,ab OR 'survey*':ti,ab OR 'health survey*':ti,ab OR 'survey methodology':ti,ab OR 'baseline survey*':ti,ab OR 'randomized response technique':ti,ab OR 'intervention study*':ti,ab OR 'psychological intervention':ti,ab OR 'psychosocial intervention':ti,ab OR 'psychological interview':ti,ab OR 'pyschophysiologic assessment':ti,ab OR 'interview':ti,ab OR 'web based intervention':ti,ab OR 'psychologic assessment':ti,ab OR 'digital intervention':ti,ab OR 'digital health intervention':ti,ab OR 'ecological momentary assessment':ti,ab OR 'institutional review':ti,ab OR 'survey methods':ti,ab OR 'respondent':ti,ab OR 'nonrespondent':ti,ab OR 'intervention':ti,ab OR 'crisis intervention':ti,ab OR 'brief treatment':ti,ab OR 'brief intervention':ti,ab OR 'critical incident stress debriefing':ti,ab OR 'psychologic interview':ti,ab OR 'assessment':ti,ab OR 'online intervention':ti,ab OR 'internet intervention':ti,ab OR 'internet based intervention':ti,ab OR 'irb':ti,ab OR 'institutional review board':ti,ab)) AND “united states”) OR (('adolescent*':ti,ab OR 'adolescence':ti,ab OR 'high school student*':ti,ab OR 'middle school student*':ti,ab OR 'minor*':ti,ab OR 'teen*':ti,ab OR 'youth*':ti,ab OR 'adolescent'/exp OR 'adolescent' OR 'adolescence'/exp OR 'adolescence' OR 'high school student'/exp OR 'high school student' OR 'middle school student'/exp OR 'middle school student' OR 'minor (person)'/exp OR 'minor (person)') AND ('mental health'/exp OR 'mental disease'/exp OR 'behavior disorder'/exp OR 'severe mental illness'/exp OR 'severe mental disorder'/exp OR 'behavioral health'/exp OR 'psychosocial rehabilitation'/exp OR 'mental health recovery'/exp OR 'psychosocial care'/exp OR 'health behavior'/exp OR 'coping behavior'/exp OR 'coping skills training'/exp OR 'adaptive behavior'/exp OR 'psychological adjustment'/exp OR 'stress management'/exp OR 'patient compliance'/exp OR 'mental health':ti,ab OR 'mental disease':ti,ab OR 'behavior disorder':ti,ab OR 'severe mental illness':ti,ab OR 'severe mental disorder':ti,ab OR 'behavioral health':ti,ab OR 'psychosocial rehabilitation':ti,ab OR 'mental health recovery':ti,ab OR 'psychosocial care':ti,ab OR 'health behavior':ti,ab OR 'coping behavior*':ti,ab OR 'coping skills training':ti,ab OR 'psychological adjustment*':ti,ab OR 'stress management':ti,ab OR 'patient compliance':ti,ab OR 'mental disorder*':ti,ab OR 'psychiatric disorder*':ti,ab OR 'psychiatric illness':ti,ab OR 'psychiatric disease*':ti,ab OR 'mental illness':ti,ab OR 'behavioral disorder*':ti,ab OR 'psychiatric diagnosis':ti,ab OR 'psychiatric rehabilitation':ti,ab OR 'mental health rehabilitation':ti,ab OR 'psychiatric health':ti,ab OR 'coping skills':ti,ab OR 'coping strategies':ti,ab OR 'adaptive behavior*':ti,ab OR 'coping':ti,ab OR 'treatment adherence':ti,ab OR 'therapeutic adherence and compliance':ti,ab OR 'therapeutic adherence':ti,ab OR 'adherence':ti,ab OR 'condition management':ti,ab) AND ('online social network'/exp OR 'online system'/exp OR 'internet'/exp OR 'mobile phone'/exp OR 'digital technology'/exp OR 'smartphone'/exp OR 'iphone'/exp OR 'blogging'/exp OR 'vlogging'/exp OR 'computer'/exp OR 'personal data assistant'/exp OR 'digital computer'/exp OR 'videoconferencing'/exp OR 'telemedicine'/exp OR 'telecare'/exp OR 'telehealth'/exp OR 'mhealth'/exp OR 'social media'/exp OR 'online survey'/exp OR 'text message'/exp OR 'text messaging'/exp OR 'short message service'/exp OR 'digital health'/exp OR 'digital health technology'/exp OR 'e-mail'/exp OR 'instant messaging'/exp OR 'tablet computer'/exp OR 'mobile application'/exp OR 'mobile health application'/exp OR 'mobile health'/exp OR 'smartphone application'/exp OR 'mobile device'/exp OR 'podcast'/exp OR 'software'/exp OR 'telemonitoring'/exp OR 'telenursing'/exp OR 'wireless communicaiton' OR 'online social network*':ti,ab OR 'online system*':ti,ab OR 'internet':ti,ab OR 'mobile phone':ti,ab OR 'digital technology':ti,ab OR 'smartphone':ti,ab OR 'iphone':ti,ab OR 'blogging':ti,ab OR 'vlogging':ti,ab OR 'computer*':ti,ab OR 'personal digital assistant':ti,ab OR 'digital computer*':ti,ab OR 'videoconferencing':ti,ab OR 'telemedicine':ti,ab OR 'telecare':ti,ab OR 'telehealth':ti,ab OR 'mhealth':ti,ab OR 'social media':ti,ab OR 'online survey':ti,ab OR 'text messag*':ti,ab OR 'short message service':ti,ab OR 'digital health':ti,ab OR 'digital health technology':ti,ab OR 'e-mail':ti,ab OR 'instant messag*':ti,ab OR 'tablet computer*':ti,ab OR 'mobile health application':ti,ab OR 'mobile health*':ti,ab OR 'mobile device':ti,ab OR 'podcast':ti,ab OR 'software':ti,ab OR 'telemonitor*':ti,ab OR 'telenursing':ti,ab OR 'wireless communication':ti,ab OR 'online':ti,ab OR 'world wide web':ti,ab OR 'website':ti,ab OR 'web based':ti,ab OR 'cell phone*':ti,ab OR 'cellular phone*':ti,ab OR 'mobile telephone':ti,ab OR 'mobile phone*':ti,ab OR 'smart phone*':ti,ab OR 'android*':ti,ab OR 'blackberr*':ti,ab OR 'web blog':ti,ab OR 'blog':ti,ab OR 'vlog':ti,ab OR 'chat room*':ti,ab OR 'chatroom*':ti,ab OR 'cyber space':ti,ab OR 'cyberspace':ti,ab OR 'digital':ti,ab OR 'internet panel':ti,ab OR 'zoom':ti,ab OR 'virtual health':ti,ab OR 'virtual medicine':ti,ab OR 'ehealth':ti,ab OR 'e health':ti,ab OR 'm health':ti,ab OR 'online stud*':ti,ab OR 'internet stud*':ti,ab OR 'text*':ti,ab OR 'digital application':ti,ab OR 'notification*':ti,ab OR 'online health':ti,ab OR 'digital mental health':ti,ab OR 'electronic mail':ti,ab OR 'email*':ti,ab OR 'ipad':ti,ab OR 'mobile app*':ti,ab OR 'smartphone app*':ti,ab OR 'palmtop computer':ti,ab OR 'palm pilot':ti,ab OR 'pocket pc':ti,ab OR 'pda*':ti,ab OR 'sms':ti,ab OR 'tablet':ti,ab OR 'web':ti,ab OR 'wireless':ti,ab OR 'wireless technology':ti,ab) AND ('questionnaire'/exp OR 'survey'/exp OR 'health survey'/exp OR 'surveys'/exp OR 'survey methodology'/exp OR 'baseline surveys'/exp OR 'randonmized response technique' OR 'intervention study'/exp OR 'crisis intervention'/exp OR 'brief intervention'/exp OR 'psychological intervention'/exp OR 'psychosocial intervention'/exp OR 'psychological interview'/exp OR 'psychophysiologic assessment'/exp OR 'interview'/exp OR 'web-based intervention'/exp OR 'psychologic assessment'/exp OR 'digital intervention'/exp OR 'digital health intervention'/exp OR 'ecological momentary assessment'/exp OR 'institutional review'/exp OR 'questionnaire*':ti,ab OR 'survey*':ti,ab OR 'health survey*':ti,ab OR 'survey methodology':ti,ab OR 'baseline survey*':ti,ab OR 'randomized response technique':ti,ab OR 'intervention study*':ti,ab OR 'psychological intervention':ti,ab OR 'psychosocial intervention':ti,ab OR 'psychological interview':ti,ab OR 'pyschophysiologic assessment':ti,ab OR 'interview':ti,ab OR 'web based intervention':ti,ab OR 'psychologic assessment':ti,ab OR 'digital intervention':ti,ab OR 'digital health intervention':ti,ab OR 'ecological momentary assessment':ti,ab OR 'institutional review':ti,ab OR 'survey methods':ti,ab OR 'respondent':ti,ab OR 'nonrespondent':ti,ab OR 'intervention':ti,ab OR 'crisis intervention':ti,ab OR 'brief treatment':ti,ab OR 'brief intervention':ti,ab OR 'critical incident stress debriefing':ti,ab OR 'psychologic interview':ti,ab OR 'assessment':ti,ab OR 'online intervention':ti,ab OR 'internet intervention':ti,ab OR 'internet based intervention':ti,ab OR 'irb':ti,ab OR 'institutional review board':ti,ab)) NOT ('canada'/exp OR 'europe'/exp OR 'asia'/exp OR 'australia and new zealand'/exp OR 'south america'/exp OR 'caribbean islands'/exp OR 'caribbean'/exp OR 'greenland'/exp OR 'south and central america'/exp OR 'mexico'/exp)) AND ('adaptive clinical trial (topic)'/de OR 'adaptive clinical trial'/de OR 'clinical trial (topic)'/de OR 'clinical trial'/de OR 'controlled clinical trial (topic)'/de OR 'controlled clinical trial'/de OR 'double blind procedure'/de OR 'early termination of clinical trial'/de OR 'equivalence trial (topic)'/de OR 'equivalence trial'/de OR 'intention to treat analysis'/de OR 'multicenter study (topic)'/de OR 'multicenter study'/de OR 'non-inferiority trial'/de OR 'phase 1 clinical trial (topic)'/de OR 'phase 1 clinical trial'/de OR 'phase 2 clinical trial (topic)'/de OR 'phase 2 clinical trial'/de OR 'phase 3 clinical trial (topic)'/de OR 'phase 3 clinical trial'/de OR 'phase 4 clinical trial (topic)'/de OR 'phase 4 clinical trial'/de OR 'pragmatic trial'/de OR 'randomized controlled trial (topic)'/de OR 'randomized controlled trial'/de OR 'superiority trial'/de OR 'multicenter study':ti,ab,kw OR 'phase i':ti,ab,kw OR 'phase ii':ti,ab,kw OR 'phase iii':ti,ab,kw OR 'phase iv':ti,ab,kw OR 'phase 1':ti,ab,kw OR 'phase 2':ti,ab,kw OR 'phase 3':ti,ab,kw OR 'phase 4':ti,ab,kw OR ((randomised OR randomized) NEAR/7 trial*) OR (controlled NEAR/3 trial*) OR (clinical NEAR/2 trial*) OR ((single:ti,ab,kw OR doubl*:ti,ab,kw OR tripl*:ti,ab,kw OR treb*:ti,ab,kw) AND (blind*:ti,ab,kw OR mask*:ti,ab,kw)) OR '4 arm':ti,ab,kw OR 'four arm':ti,ab,kw)) AND (2020:py OR 2021:py OR 2022:py OR 2023:py OR 2024:py OR 2025:py OR 2026:py) |
| PsycInfo | [((((Tiab("adolescent" OR "adolescence" OR "teenager" OR "teen" OR "youth" OR "high school" OR "middle school" OR "minor" OR "minors") OR MAINSUBJECT.EXACT("Middle School Students") OR MAINSUBJECT.EXACT("High School Students")) AND (Tiab("mental health" OR "mental disorder" OR "psychiatric disorder" OR "psychiatric illness" OR "psychiatric disease" OR "mental illness" OR "behavioral disorder" OR "psychiatric diagnosis" OR "psychiatric rehabilitation" OR "severe mental illness" OR "severe mental disorder" OR "behavioral health" OR "psychosocial rehabilitation" OR "mental health recovery" OR "psychiatric rehabilitation" OR "mental health rehabilitation" OR "psychosocial care" OR "psychiatric health" OR "health behavior" OR "coping behavior" OR "coping skills" OR "coping strategies" OR "adaptive behaviors" OR "coping" OR "stress management" OR "treatment adherence" OR "therapeutic adherence and compliance" OR "therapeutic adherence" OR "adherence" OR "condition management" OR "mental disease" OR "behavior disorder" OR "patient compliance" OR "psychological adjustment") OR MAINSUBJECT.EXACT("Psychosocial Rehabilitation") OR MAINSUBJECT.EXACT("Adaptive Behavior") OR MAINSUBJECT.EXACT("Health Risk Behavior") OR MAINSUBJECT.EXACT("Disruptive Behavior Disorders") OR MAINSUBJECT.EXACT("Stress Management") OR MAINSUBJECT.EXACT("Health Behavior") OR MAINSUBJECT.EXACT("Emotional and Behavioral Disorders") OR MAINSUBJECT.EXACT("Health Behavior Measures") OR MAINSUBJECT.EXACT("Treatment Compliance") OR MAINSUBJECT.EXACT("Behavioral Health Services") OR MAINSUBJECT.EXACT("Psychosocial Readjustment") OR MAINSUBJECT.EXACT("Coping Behavior") OR MAINSUBJECT.EXACT("Behavior Disorders") OR MAINSUBJECT.EXACT.EXPLODE("Mental Health") OR MAINSUBJECT.EXACT("Emotional and Behavioral Disorders") OR MAINSUBJECT.EXACT("Mental Disorders") OR MAINSUBJECT.EXACT("Serious Mental Illness")) OR MAINSUBJECT.EXACT("Adolescent Psychology") OR MAINSUBJECT.EXACT("Adolescent Psychiatry") OR MAINSUBJECT.EXACT("Adolescent Psychotherapy") OR MAINSUBJECT.EXACT("Youth Mental Health")) AND (TIAB("online social networking" OR "online systems" OR "online" OR "internet" OR "world wide web" OR "mobile phone" OR "cell phone" OR "cellular phone" OR "mobile telephone" OR "smartphone" OR "smart phone" OR "android" OR "blackberry" OR "website" OR "web based" OR "digital technology" OR "iphone" OR "web blog" OR "blog" OR "vlog" OR "chat room" OR "chatroom" OR "blogging" OR "vlogging" OR "computer" OR "cyber space" OR "cyberspace" OR "digital" OR "digital computer" OR "internet panel" OR "zoom" OR "videoconferencing" OR "videoconference" OR "telemedicine" OR "virtual health" OR "virtual medicine" OR "ehealth" OR "e health" OR "mhealth" OR "m health" OR "online studies" OR "internet studies" OR "social media" OR "online survey" OR "text messaging" OR "short message service" OR "text message" OR "text" OR "digital application" OR "notification" OR "digital health" OR "digital health technology" OR "online health" OR "digital mental health" OR "electronic mail" OR "email" OR "e-mail" OR "instant message" OR "instant messaging" OR "instant messages" OR "instant messenger" OR "ipad" OR "mobile application" OR "mobile app" OR "mobile health" OR "smartphone app" OR "smartphone application" OR "mobile healthcare" OR "tablet computer" OR "palmtop computer" OR "palm pilot" OR "pocket PC" OR "PDA" OR "PDAs" OR "personal digital assistant" OR "mobile device" OR "podcast" OR "SMS" OR "software" OR "tablet" OR "telecare" OR "telehealth" OR "telemonitor" OR "telemonitoring" OR "telenursing" OR "web" OR "wireless technology" OR "wireless" OR "wireless communication" OR "mobile health application") OR MAINSUBJECT.EXACT("Online Social Networks") OR MAINSUBJECT.EXACT("Internet") OR MAINSUBJECT.EXACT("Mobile Technology") OR MAINSUBJECT.EXACT.EXPLODE("Mobile Phones") OR MAINSUBJECT.EXACT.EXPLODE("Mobile Technology") OR MAINSUBJECT.EXACT("Mobile Applications") OR MAINSUBJECT.EXACT("Websites") OR MAINSUBJECT.EXACT("Computer Software") OR MAINSUBJECT.EXACT("Computer Mediated Communication") OR MAINSUBJECT.EXACT("Digital Mental Health Resources") OR MAINSUBJECT.EXACT("Text Messaging") OR MAINSUBJECT.EXACT("Smartphones") OR MAINSUBJECT.EXACT.EXPLODE("Telemedicine") OR MAINSUBJECT.EXACT("Digital Computers") OR MAINSUBJECT.EXACT("Social Media") OR MAINSUBJECT.EXACT("Wireless Technologies") OR MAINSUBJECT.EXACT("Computers") OR MAINSUBJECT.EXACT("Telepsychiatry") OR MAINSUBJECT.EXACT("Online Therapy") OR MAINSUBJECT.EXACT("Digital Health Resources") OR MAINSUBJECT.EXACT("Laptop Computers") OR MAINSUBJECT.EXACT("Digital Technology")) AND (TIAB("survey" OR "survey" OR "survey methods" OR "survey methodology" OR "baseline survey" OR "respondent" OR "nonrespondent" OR "randomized response technique" OR "intervention" OR "crisis intervention" OR "brief treatment" OR "brief intervention" OR "critical incident stress debriefing" OR "psychosocial intervention" OR "psychological intervention" OR "psycholphysiological assessment" OR "psychological interview" OR "psychologic interview" OR "questionnaire" OR "interview" OR "internet based intervention" OR "assessment" OR "online intervention" OR "internet intervention" OR "web based intervention" OR "digital intervention" OR "digital health intervention" OR "ecological momentary assessment" OR "IRB" OR "institutional review board" OR "institutional review" OR "intervention study" OR "health survey" OR "psychologic assessment") OR MAINSUBJECT.EXACT("Interviews") OR MAINSUBJECT.EXACT("Questionnaires") OR MAINSUBJECT.EXACT("Brief Interventions") OR MAINSUBJECT.EXACT("Crisis Intervention") OR MAINSUBJECT.EXACT("Health Psychology Assessment") OR MAINSUBJECT.EXACT("Cognitive Interview") OR MAINSUBJECT.EXACT("Behavioral Assessment") OR MAINSUBJECT.EXACT("Mental Health and Illness Assessment") OR MAINSUBJECT.EXACT("Ecological Momentary Assessment") OR MAINSUBJECT.EXACT("Psychosocial Rehabilitation") OR MAINSUBJECT.EXACT("Interviewing") OR MAINSUBJECT.EXACT("Electronic Health Services") OR MAINSUBJECT.EXACT("Consumer Surveys") OR MAINSUBJECT.EXACT("Intervention") OR MAINSUBJECT.EXACT("Cognitive Assessment") OR MAINSUBJECT.EXACT("Surveys") OR MAINSUBJECT.EXACT("Online Surveys") OR MAINSUBJECT.EXACT("Psychosocial Assessment") OR MAINSUBJECT.EXACT("Digital Interventions") OR MAINSUBJECT.EXACT("Debriefing (Psychological)") OR MAINSUBJECT.EXACT("Psychological Assessment") OR MAINSUBJECT.EXACT("Psychosocial Readjustment")) AND (United States)) OR (((Tiab("adolescent" OR "adolescence" OR "teenager" OR "teen" OR "youth" OR "high school" OR "middle school" OR "minor" OR "minors") OR MAINSUBJECT.EXACT("Middle School Students") OR MAINSUBJECT.EXACT("High School Students")) AND (Tiab("mental health" OR "mental disorder" OR "psychiatric disorder" OR "psychiatric illness" OR "psychiatric disease" OR "mental illness" OR "behavioral disorder" OR "psychiatric diagnosis" OR "psychiatric rehabilitation" OR "severe mental illness" OR "severe mental disorder" OR "behavioral health" OR "psychosocial rehabilitation" OR "mental health recovery" OR "psychiatric rehabilitation" OR "mental health rehabilitation" OR "psychosocial care" OR "psychiatric health" OR "health behavior" OR "coping behavior" OR "coping skills" OR "coping strategies" OR "adaptive behaviors" OR "coping" OR "stress management" OR "treatment adherence" OR "therapeutic adherence and compliance" OR "therapeutic adherence" OR "adherence" OR "condition management" OR "mental disease" OR "behavior disorder" OR "patient compliance" OR "psychological adjustment") OR MAINSUBJECT.EXACT("Psychosocial Rehabilitation") OR MAINSUBJECT.EXACT("Adaptive Behavior") OR MAINSUBJECT.EXACT("Health Risk Behavior") OR MAINSUBJECT.EXACT("Disruptive Behavior Disorders") OR MAINSUBJECT.EXACT("Stress Management") OR MAINSUBJECT.EXACT("Health Behavior") OR MAINSUBJECT.EXACT("Emotional and Behavioral Disorders") OR MAINSUBJECT.EXACT("Health Behavior Measures") OR MAINSUBJECT.EXACT("Treatment Compliance") OR MAINSUBJECT.EXACT("Behavioral Health Services") OR MAINSUBJECT.EXACT("Psychosocial Readjustment") OR MAINSUBJECT.EXACT("Coping Behavior") OR MAINSUBJECT.EXACT("Behavior Disorders") OR MAINSUBJECT.EXACT.EXPLODE("Mental Health") OR MAINSUBJECT.EXACT("Emotional and Behavioral Disorders") OR MAINSUBJECT.EXACT("Mental Disorders") OR MAINSUBJECT.EXACT("Serious Mental Illness")) OR MAINSUBJECT.EXACT("Adolescent Psychology") OR MAINSUBJECT.EXACT("Adolescent Psychiatry") OR MAINSUBJECT.EXACT("Adolescent Psychotherapy") OR MAINSUBJECT.EXACT("Youth Mental Health")) AND (TIAB("online social networking" OR "online systems" OR "online" OR "internet" OR "world wide web" OR "mobile phone" OR "cell phone" OR "cellular phone" OR "mobile telephone" OR "smartphone" OR "smart phone" OR "android" OR "blackberry" OR "website" OR "web based" OR "digital technology" OR "iphone" OR "web blog" OR "blog" OR "vlog" OR "chat room" OR "chatroom" OR "blogging" OR "vlogging" OR "computer" OR "cyber space" OR "cyberspace" OR "digital" OR "digital computer" OR "internet panel" OR "zoom" OR "videoconferencing" OR "videoconference" OR "telemedicine" OR "virtual health" OR "virtual medicine" OR "ehealth" OR "e health" OR "mhealth" OR "m health" OR "online studies" OR "internet studies" OR "social media" OR "online survey" OR "text messaging" OR "short message service" OR "text message" OR "text" OR "digital application" OR "notification" OR "digital health" OR "digital health technology" OR "online health" OR "digital mental health" OR "electronic mail" OR "email" OR "e-mail" OR "instant message" OR "instant messaging" OR "instant messages" OR "instant messenger" OR "ipad" OR "mobile application" OR "mobile app" OR "mobile health" OR "smartphone app" OR "smartphone application" OR "mobile healthcare" OR "tablet computer" OR "palmtop computer" OR "palm pilot" OR "pocket PC" OR "PDA" OR "PDAs" OR "personal digital assistant" OR "mobile device" OR "podcast" OR "SMS" OR "software" OR "tablet" OR "telecare" OR "telehealth" OR "telemonitor" OR "telemonitoring" OR "telenursing" OR "web" OR "wireless technology" OR "wireless" OR "wireless communication" OR "mobile health application") OR MAINSUBJECT.EXACT("Online Social Networks") OR MAINSUBJECT.EXACT("Internet") OR MAINSUBJECT.EXACT("Mobile Technology") OR MAINSUBJECT.EXACT.EXPLODE("Mobile Phones") OR MAINSUBJECT.EXACT.EXPLODE("Mobile Technology") OR MAINSUBJECT.EXACT("Mobile Applications") OR MAINSUBJECT.EXACT("Websites") OR MAINSUBJECT.EXACT("Computer Software") OR MAINSUBJECT.EXACT("Computer Mediated Communication") OR MAINSUBJECT.EXACT("Digital Mental Health Resources") OR MAINSUBJECT.EXACT("Text Messaging") OR MAINSUBJECT.EXACT("Smartphones") OR MAINSUBJECT.EXACT.EXPLODE("Telemedicine") OR MAINSUBJECT.EXACT("Digital Computers") OR MAINSUBJECT.EXACT("Social Media") OR MAINSUBJECT.EXACT("Wireless Technologies") OR MAINSUBJECT.EXACT("Computers") OR MAINSUBJECT.EXACT("Telepsychiatry") OR MAINSUBJECT.EXACT("Online Therapy") OR MAINSUBJECT.EXACT("Digital Health Resources") OR MAINSUBJECT.EXACT("Laptop Computers") OR MAINSUBJECT.EXACT("Digital Technology")) AND (TIAB("survey" OR "survey" OR "survey methods" OR "survey methodology" OR "baseline survey" OR "respondent" OR "nonrespondent" OR "randomized response technique" OR "intervention" OR "crisis intervention" OR "brief treatment" OR "brief intervention" OR "critical incident stress debriefing" OR "psychosocial intervention" OR "psychological intervention" OR "psycholphysiological assessment" OR "psychological interview" OR "psychologic interview" OR "questionnaire" OR "interview" OR "internet based intervention" OR "assessment" OR "online intervention" OR "internet intervention" OR "web based intervention" OR "digital intervention" OR "digital health intervention" OR "ecological momentary assessment" OR "IRB" OR "institutional review board" OR "institutional review" OR "intervention study" OR "health survey" OR "psychologic assessment") OR MAINSUBJECT.EXACT("Interviews") OR MAINSUBJECT.EXACT("Questionnaires") OR MAINSUBJECT.EXACT("Brief Interventions") OR MAINSUBJECT.EXACT("Crisis Intervention") OR MAINSUBJECT.EXACT("Health Psychology Assessment") OR MAINSUBJECT.EXACT("Cognitive Interview") OR MAINSUBJECT.EXACT("Behavioral Assessment") OR MAINSUBJECT.EXACT("Mental Health and Illness Assessment") OR MAINSUBJECT.EXACT("Ecological Momentary Assessment") OR MAINSUBJECT.EXACT("Psychosocial Rehabilitation") OR MAINSUBJECT.EXACT("Interviewing") OR MAINSUBJECT.EXACT("Electronic Health Services") OR MAINSUBJECT.EXACT("Consumer Surveys") OR MAINSUBJECT.EXACT("Intervention") OR MAINSUBJECT.EXACT("Cognitive Assessment") OR MAINSUBJECT.EXACT("Surveys") OR MAINSUBJECT.EXACT("Online Surveys") OR MAINSUBJECT.EXACT("Psychosocial Assessment") OR MAINSUBJECT.EXACT("Digital Interventions") OR MAINSUBJECT.EXACT("Debriefing (Psychological)") OR MAINSUBJECT.EXACT("Psychological Assessment") OR MAINSUBJECT.EXACT("Psychosocial Readjustment")) NOT (Canada OR Mexico OR Greenland OR "South America" OR "Central America" OR Europe OR Asia OR Australia OR "Caribbean Islands")) AND (exp Clinical Trials/ OR Placebo/ OR (random* OR sham OR placebo* OR ((singl* OR doubl*) adj (blind* OR dumm* OR mask*)) OR ((tripl* OR trebl*) adj (blind* OR dumm* OR mask*)) OR (control* adj3 (study OR studies OR trial* OR group*)) OR Nonrandom* OR non random* OR non-random* OR quasi-random* OR quasirandom* OR allocated OR ((open label OR open-label) adj5 (study OR studies OR trial*)) OR ((equivalence OR superiority OR non-inferiority OR noninferiority) adj3 (study OR studies OR trial*)) OR ((pragmatic OR practical) adj3 trial*) OR ((quasiexperimental OR quasi-experimental) adj3 (study OR studies OR trial*)) OR (phase adj3 (III OR "3") adj3 (study OR studies OR trial*))) .ti,ab,hw.)) AND pd(20200101-20260617)](https://www.proquest.com/myresearch/savedsearches.checkdbssearchlink:rerunsearch/2585281/SavedSearches/$N?_csrf=625df3cd-199f-4ff1-9795-ab3845e3cecb&site=psycinfo&t:ac=SavedSearches) |
| ACM Digital Library | [[Abstract: "adolescent"] OR [Abstract: "adolescence"] OR [Abstract: "teenager*"] OR [Abstract: "teen*"] OR [Abstract: "youth*"] OR [Abstract: "high school"] OR [Abstract: "middle school"] OR [Abstract: "minor*"]] AND [[Abstract: "mental health"] OR [Abstract: "psychiatric disorder"] OR [Abstract: "psychiatric illness"] OR [Abstract: "psychiatric disease"] OR [Abstract: "mental illness"] OR [Abstract: "behavioral disorder"] OR [Abstract: "psychiatric diagnosis"] OR [Abstract: "severe mental illness"] OR [Abstract: "severe mental disorder"] OR [Abstract: "mental disorder"] OR [Abstract: "behavioral health"] OR [Abstract: "psychiatric rehabilitation"] OR [Abstract: "psychosocial rehabilitation"] OR [Abstract: "mental health recovery"] OR [Abstract: "mental health rehabilitation"] OR [Abstract: "psychosocial care"] OR [Abstract: "psychiatric health"] OR [Abstract: "health behavior"] OR [Abstract: "coping behavior"] OR [Abstract: "coping skills"] OR [Abstract: "coping strategies"] OR [Abstract: "adaptive behaviors"] OR [Abstract: "coping"] OR [Abstract: "stress management"] OR [[Abstract: "therapeutic adherence and compliance"] AND [Abstract: "adherence"]] OR [Abstract: "condition management"] OR [Abstract: "mental disease"] OR [Abstract: "behavior disorder"] OR [Abstract: "patient compliance"] OR [Abstract: "psychological adjustment"]] AND [[Abstract: "online"] OR [Abstract: "internet"] OR [Abstract: "website"] OR [Abstract: "cell phone"] OR [Abstract: "cellular phone"] OR [Abstract: "mobile telephone"] OR [Abstract: "mobile phone"] OR [Abstract: "digital technology"] OR [Abstract: "smartphone"] OR [Abstract: "smart phone"] OR [Abstract: "android*"] OR [Abstract: "blackberr*"] OR [Abstract: "iphone*"] OR [[Abstract: "web blog"] AND [Abstract: "blog*"]] OR [Abstract: "vlog*"] OR [Abstract: "chat room"] OR [Abstract: "chat rooms"] OR [Abstract: "chatroom*"] OR [Abstract: "computer*"] OR [Abstract: "cyber space"] OR [Abstract: "cyberspace"] OR [Abstract: "digital"] OR [Abstract: "zoom"] OR [Abstract: "videoconferenc*"] OR [Abstract: "telemedicine"] OR [Abstract: "virtual health"] OR [Abstract: "virtual medicine"] OR [Abstract: "ehealth"] OR [Abstract: "e health"] OR [Abstract: "mhealth"] OR [Abstract: "m health"] OR [Abstract: "online studies"] OR [Abstract: "internet studies"] OR [Abstract: "social media"] OR [Abstract: "short message service"] OR [Abstract: "text*"] OR [Abstract: "digital application"] OR [Abstract: "notification*"] OR [Abstract: "digital health"] OR [Abstract: "digital health technology"] OR [Abstract: "online health"] OR [Abstract: "digital mental health"] OR [Abstract: "electronic mail"] OR [Abstract: "email*"] OR [Abstract: "e-mail*"] OR [Abstract: "instant message"] OR [Abstract: "instant messaging"] OR [Abstract: "instant messages"] OR [Abstract: "instant messenger"] OR [Abstract: "ipad*"] OR [Abstract: "mobile application*"] OR [Abstract: "mobile app"] OR [Abstract: "mobile health"] OR [Abstract: "smartphone app"] OR [Abstract: "smartphone application"] OR [Abstract: "mobile healthcare"] OR [Abstract: "computers, handheld"] OR [Abstract: "palmtop computer"] OR [Abstract: "palm pilot"] OR [Abstract: "pocket pc"] OR [Abstract: "pda"] OR [Abstract: "pdas"] OR [Abstract: "personal digital assistant"] OR [Abstract: "mobile device"] OR [Abstract: "podcast*"] OR [Abstract: "sms"] OR [Abstract: "software"] OR [Abstract: "tablet*"] OR [Abstract: "telecare"] OR [Abstract: "telehealth"] OR [Abstract: "telemonitor*"] OR [Abstract: "telenursing"] OR [Abstract: "web"] OR [Abstract: "wireless"] OR [Abstract: "blogging"] OR [Abstract: "vlogging"] OR [Abstract: "mobile health application"]] AND [[Abstract: "survey*"] OR [Abstract: "respondent*"] OR [Abstract: "randomized response technique"] OR [Abstract: "nonrespondent*"] OR [Abstract: "intervention"] OR [Abstract: "crisis intervention"] OR [Abstract: "brief treatment"] OR [Abstract: "brief intervention"] OR [Abstract: "critical incident stress debriefing"] OR [Abstract: "psychosocial intervention"] OR [Abstract: "psychological intervention"] OR [Abstract: "questionnaire*"] OR [Abstract: "interview*"] OR [Abstract: "internet-based intervention"] OR [Abstract: "assessment*"] OR [Abstract: "online intervention"] OR [Abstract: "internet intervention"] OR [Abstract: "internet based intervention"] OR [Abstract: "web based intervention"] OR [Abstract: "digital intervention"] OR [Abstract: "digital health intervention"] OR [Abstract: "ecological momentary assessment"] OR [Abstract: "irb"] OR [Abstract: "institutional review board"] OR [Abstract: "institutional review"] OR [Abstract: "intervention study"] OR [Abstract: "health survey"] OR [Abstract: "psychologic assessment"]] AND [E-Publication Date: (01/01/2020 TO 06/17/2026)] |
|  |  |
